# Crystal Structure Based Mutagenesis of Cattleyene Synthase Leads to the Generation of Rearranged Polycyclic Diterpenes

**DOI:** 10.1002/anie.202209785

**Published:** 2022-08-01

**Authors:** Baiying Xing, Houchao Xu, Annan Li, Tingting Lou, Meng Xu, Kaibiao Wang, Zhengren Xu, Jeroen S. Dickschat, Donghui Yang, Ming Ma

**Affiliations:** ^1^ State Key Laboratory of Natural and Biomimetic Drugs School of Pharmaceutical Sciences Peking University 38 Xueyuan Road, Haidian District Beijing 100191 China; ^2^ Kekulé-Institute for Organic Chemistry and Biochemistry University of Bonn Gerhard-Domagk-Strasse 1 53121 Bonn Germany

**Keywords:** Biosynthesis, Diterpenes, Enzymes, Natural Products, Reaction Mechanisms

## Abstract

The crystal structures of cattleyene synthase (apo‐CyS), and CyS complexed with geranylgeranyl pyrophosphate (GGPP) were solved. The CyS^C59A^ variant exhibited an increased production of cattleyene and other diterpenes with diverse skeletons. Its structure showed a widened active site cavity explaining the relaxed selectivity. Isotopic labeling experiments revealed a remarkable cyclization mechanism involving several skeletal rearrangements for one of the novel diterpenes.

## Graphical abstract

The crystal structure of the diterpene synthase CyS was solved, providing the structural basis for cattleyene biosynthesis. A CyS^C59A^ mutant generated diterpenes with novel carbon skeletons. The remarkable cyclization mechanism of one of these products was characterized by isotopic labeling experiments.

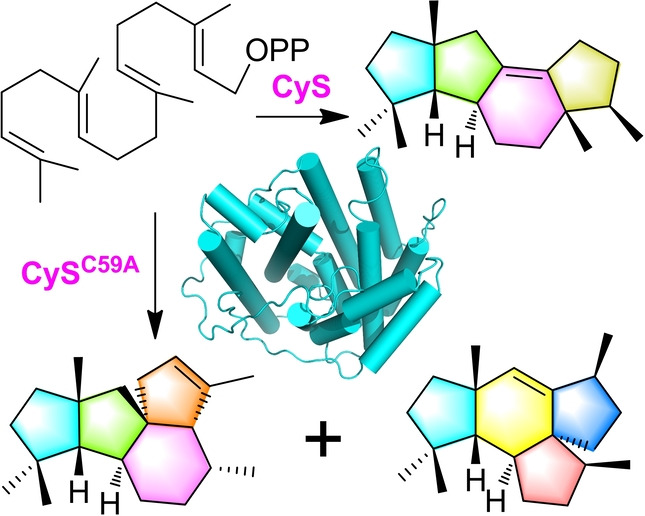

Diterpenoids exhibit diverse chemical skeletons and important biological activities.^[1]^ Because of the larger number of possible reactions for geranylgeranyl pyrophosphate (GGPP) as compared to geranyl (GPP) and farnesyl pyrophosphate (FPP), diterpenoids usually exhibit more complex skeletons than mono‐ and sesquiterpenoids (Figure 1A). Some polycyclic diterpenoids have attracted increasing attention, e.g. gibberellins are phytohormones derived from *ent*‐kaurene,^[2]^ while phorbol esters exhibiting the tigliane skeleton are currently in phase II clinical trials for the treatment of acute myeloid leukemia (Figure 1A).[^3^, ^4^] Their biosynthesis is attributed to diterpene synthases (DTSs), the type I of which catalyzes the conversion of GGPP through diphosphate abstraction and cationic cascade reactions. Substrate ionization is mediated by a Lewis acidic trinuclear Mg^2+^ cluster, bound itself to a highly conserved Asp‐rich motif (DDXX(X)D) and an NSE/DTE triad ((N,D)DLX(S,T)XXXE), to which the substrate's pyrophosphate docks,^[5]^ with assistance of a highly conserved Arg residue (pyrophosphate sensor).^[6]^ A main chain carbonyl oxygen in the effector triad is involved in the stabilization of the initially formed allyl cation^[6]^ and serves as a catalytic base and acid in the formation and reprotonation of neutral intermediates.^[7]^ The mechanisms of these multistep processes can be probed by isotopic labeling experiments,^[8]^ revealing an astonishing complexity associated with a single enzymatic transformation. This enzymatic power is often superior to the long and laborious routes^[9]^ with low overall yields^[10]^ to diterpenes by chemical synthesis. Several DTSs with structurally complex products have been reported,[^11^, ^12^, ^13^, ^14^, ^15^, ^16^, ^17^, ^18^, ^19^, ^20^] but only a few structures of class I DTSs have been solved, including taxadiene synthase from *Taxus brevifolia*,^[21]^ spiroviolene synthase (SvS) from *Streptomyces violens* ^[22]^ and cyclooctat‐9‐en‐7‐ol synthase (CotB2) from *S. melanosporofaciens*,[^23^, ^24^, ^25^] *ent*‐kaurene synthase from *Bradyrhizobium japonicum*,^[26]^ and isopimarane synthases Sat1646 from *Salinispora* sp. and Stt4548 from *Streptomyces* sp. (Figure 1A).^[27]^ Structural knowledge of DTSs is of interest to deepen our mechanistic understanding of these enzymes and allows for structure based site‐directed mutagenesis.[^25^, ^28^, ^29^]

**Figure 1:**
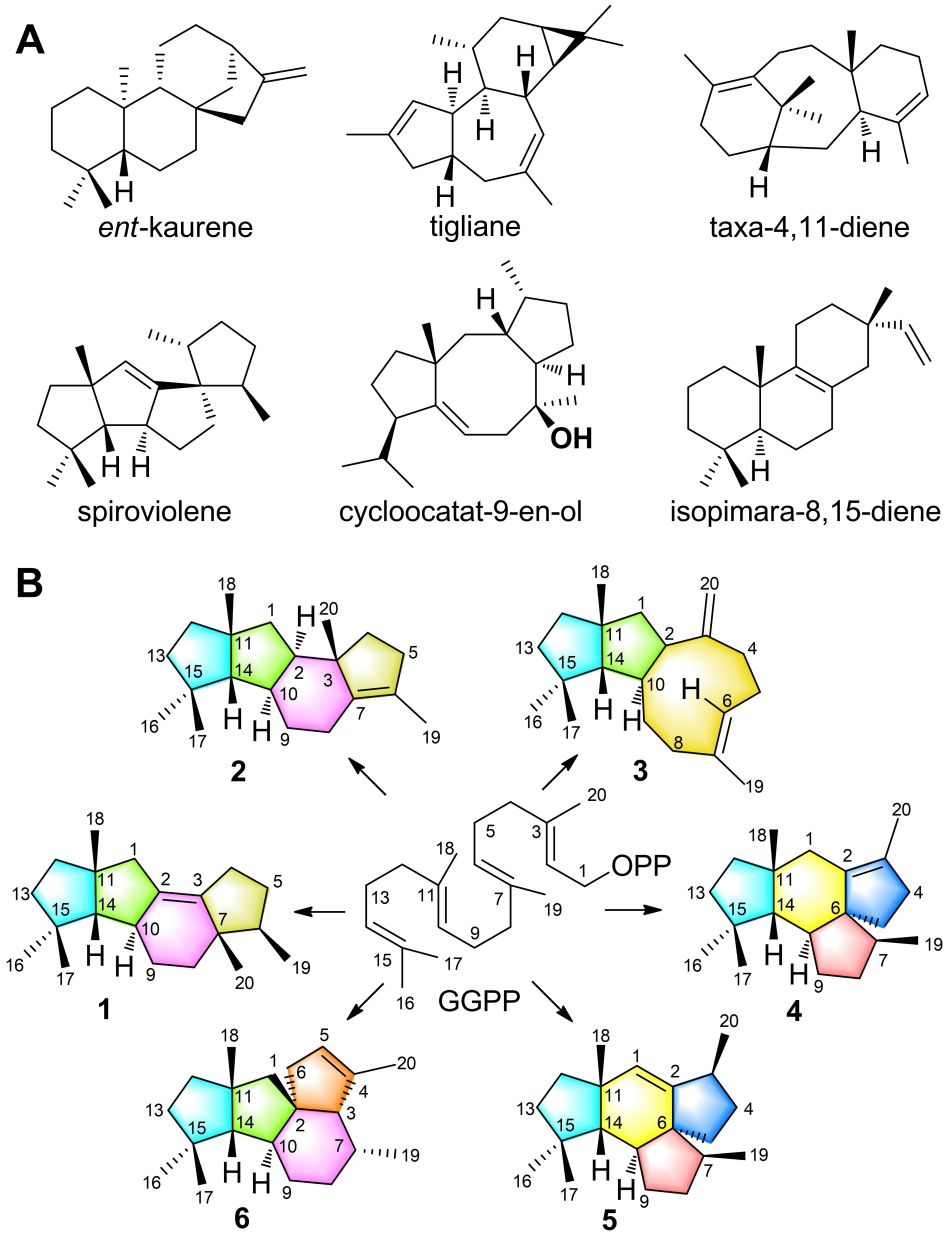
Representative diterpenes. A) Compounds from previous studies, B) products of CyS and its variants.

We recently discovered the cattleyene (**1**) synthase (CyS) from *Streptomyces cattleya* (Figure 1B) and studied its cyclization mechanism through isotopic labelings.^[18]^ Here we report on the crystal structures of apo‐CyS, CyS complexed with GGPP and Mg^2+^ (CyS‐GGPP‐Mg^2+^), and the CyS^C59A^ enzyme variant. Modellings in conjunction with site‐directed mutagenesis and additional labeling experiments are discussed that provide a deeper understanding of cattleyene production by CyS.

High‐quality crystals of purified CyS (Figure S1) were obtained and the structure of apo‐CyS was solved at 2.00 Å, using the structure of SvS^[22]^ as template (PDB ID: 6TBD). Crystals of CyS‐GGPP‐Mg^2+^ were obtained by soaking and the structure was solved at 1.87 Å using the apo‐CyS structure as template. CyS‐GGPP‐Mg^2+^ is the first structure of a terpene synthase (TS) in complex with the native substrate GGPP, providing an ideal opportunity for analyzing GGPP binding and interactions in the active site.

Apo‐CyS adopts the classical α‐helical fold of class I TSs, with ten core (A‐J) and three short α helices (α1–α3, Figure 2A). The Asp‐rich motif (D^89^DVHCD^94^) is located on helix C and the NSE triad (D^232^DLFS^236^YGKE^240^) on helix H. The CyS‐GGPP‐Mg^2+^ structure shows a similar fold to apo‐CyS (Figure 2B), with a root‐mean‐square deviation (RMSD) of 0.24 Å for Cα atoms. Upon GGPP and Mg^2+^ binding one more α helix (α4) and two β strands (β1 and β2) close to the active site become ordered (Figure 2C). Further differences are observed for residues involved in Mg^2+^‐binding (Figure 2D), but only two Mg^2+^ ions are found (Mg^2+^_C_ coordinated by R324, D89 and D90, and Mg^2+^_B_ coordinated by R186, N232 and S236; Figures 2D and S1B), while Mg^2+^_A_ as observed in selinadiene synthase^[6]^ is missing. The pyrophosphate moiety of GGPP binds to both Mg^2+^ and the conserved C‐terminal RY (R324 and Y325).

**Figure 2:**
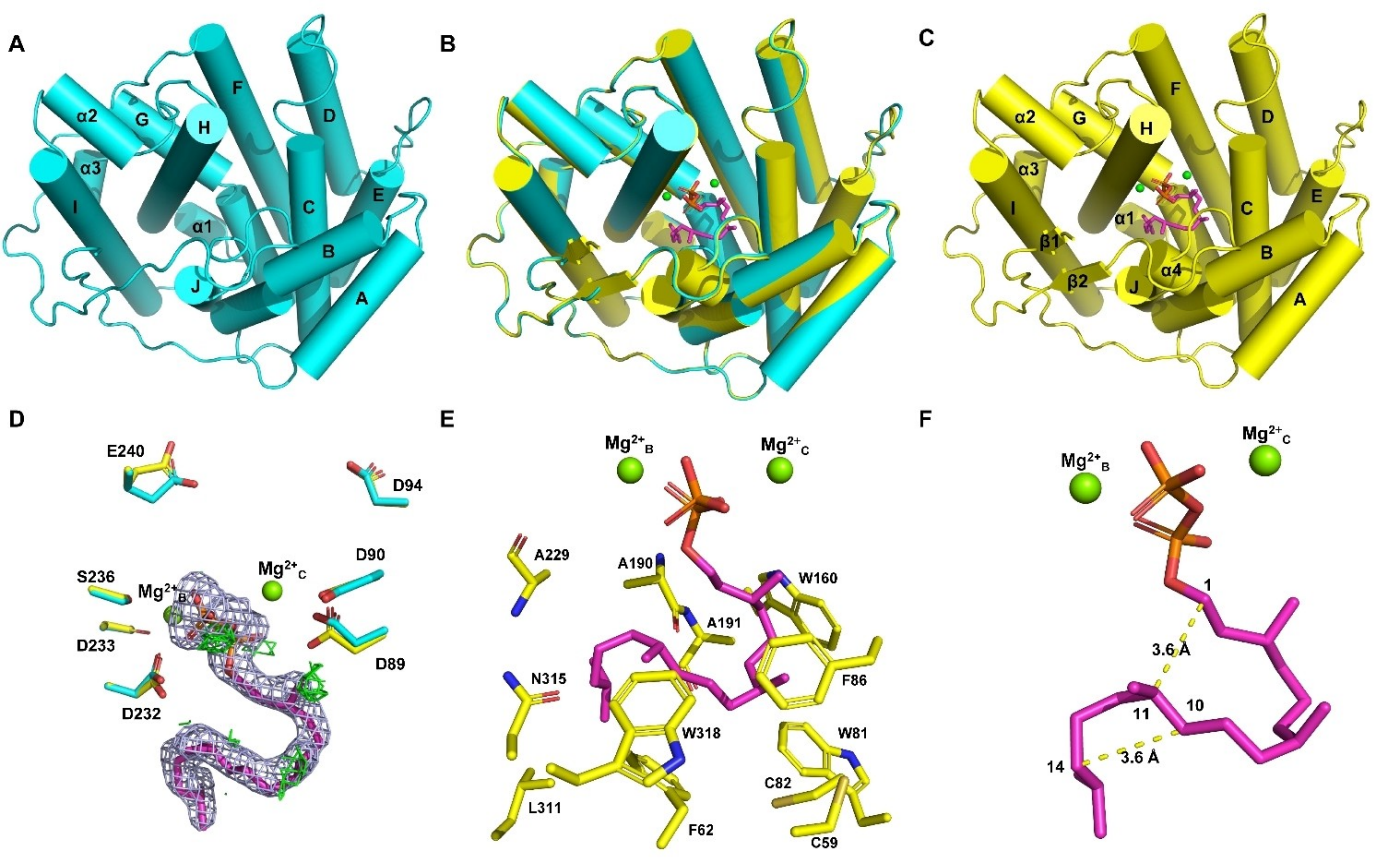
Crystal structures of A) apo‐CyS, B) superimposition of apo‐CyS and CyS‐GGPP‐Mg^2+^, and C) CyS‐GGPP‐Mg^2+^. D) Superimposition of aspartate‐rich motifs in apo‐CyS (cyan) and CyS‐GGPP‐Mg^2+^ (yellow). The 2 *F*_o_−*F*_c_ and *F*_o_−*F*_c_ electron density maps of GGPP, contoured at 2*σ*, are shown as light blue and green meshes (only positive densities are found). E) Residues surrounding GGPP. F) Conformation of GGPP in the active site. Mg^2+^ ions are shown in green, GGPP is shown in magenta.

GGPP is surrounded by five aromatic (F62, W81, F86, W160, and W318), four aliphatic (A190, A191, A229, and L311) and three polar residues (C59, C82, and N315, Figure 2E). These interactions render GGPP folded into a specific conformation, in which C‐11 and C‐14 are close to C‐1 and C‐10 with both distances of 3.6 Å (Figure 2F), allowing the formation of the 5/11 bicyclic intermediate in the first cyclization steps. The GGPP conformation is P‐helical from C‐1 to C‐11 and M‐helical from C‐10 to C‐14, which explains the observed stereoselectivity of the C‐1/C‐11 and C‐10/C‐14 bond formations to generate the 10*S*, 11*R* and 14*S* configurations.

Key intermediates were modelled into the CyS active site using CyS‐GGPP‐Mg^2+^ as the macromolecule in AutoDock Vina 1.1.2, by removing water and the geranylgeranyl moiety of GGPP. Intermediates **A**–**H** were prepared by using default parameters (Supporting Information). The intermediates **A**–**G** are stabilized by cation‐π interactions and van der Waals forces with aromatic residues (F62, W318, W81, F86, and W160, Figure 3). The last intermediate **H** is stabilized by a cation‐dipole interaction with the A190 main chain carbonyl group (effector), in an equivalent position to the effector G182 in selinadiene synthase.^[6]^ Notably, no polar residue or water is found near the cation at C‐3, but the pyrophosphate is only 4.1 Å away from C‐2 and may abstract the C‐2 proton to form **1** (Figure 3).^[18]^

**Figure 3:**
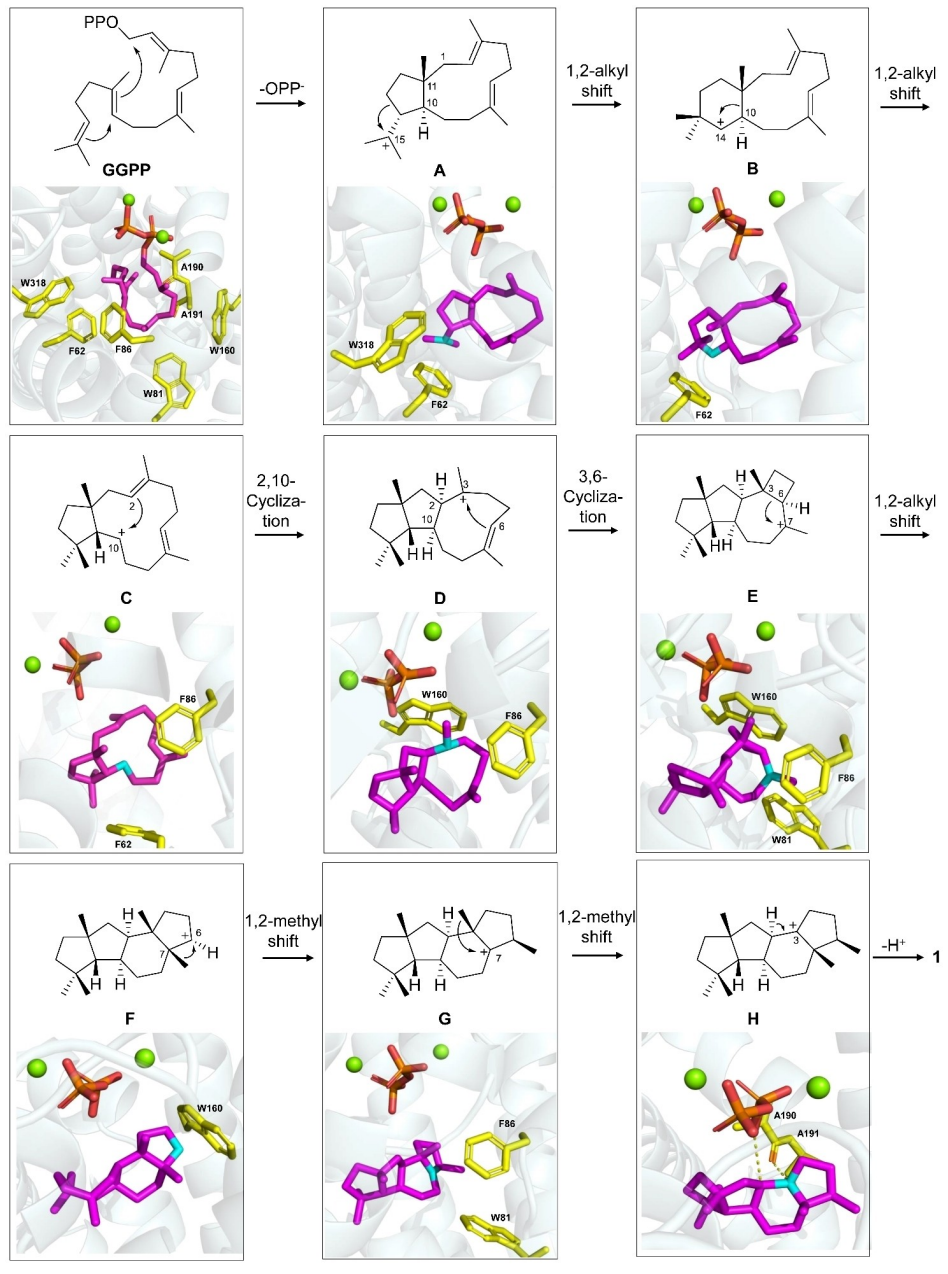
Biosynthesis of **1** and modelling of intermediates into the active site of CyS. Colour code: Active site residues (yellow), geranylgeranyl chain and intermediates **A**–**H** (magenta, cationic centers in cyan), diphosphate (red), and Mg^2+^ ions (green). A previously suggested mechanism avoids secondary cation **B** by a concerted mechanism from **A** to **C**.[^13^, ^18^]

The above modellings show how CyS catalyzes the biosynthesis of **1**. The roles of the identified key residues were then investigated by site‐directed mutagenesis (Figure 4). For this purpose, an engineered *E. coli* strain containing a reconstructed isopentenol utilization pathway (IUP) to produce isopentenyl (IPP) and dimethylallyl pyrophosphate (DMAPP) was used (Figure S2). The F62A and W318A enzyme variants showed a substantially decreased or completely abolished production of **1**, confirming the important roles of F62 and W318 in the biosynthesis of **1**. The W81A variant only produced minor amounts of **1**. Considering the interaction of W81 with GGPP, this residue may be required to keep the substrate and the intermediates in suitable conformations.

**Figure 4:**
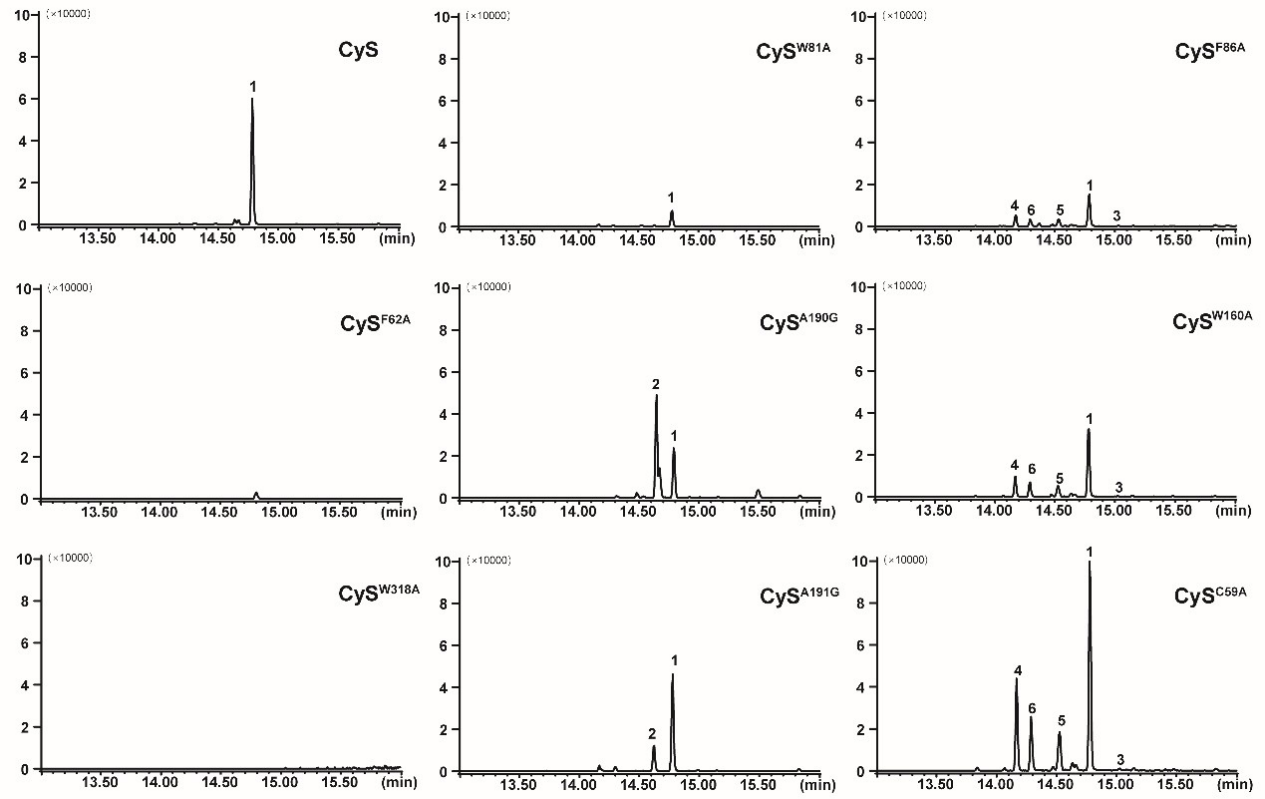
GC‐MS analysis (extracted ion chromatograms at *m*/*z* 272) of products by CyS and its variants.

The variations of A190G and A191G caused the formation of an additional product **2**, while the exchanges of F86A and W160A resulted in decreased levels of **1** with simultaneous production of several new compounds (**3**–**6**). Diterpene **3** was only observed in trace amounts, and yields of **4**, **5** and **6** were about 30 %, 20 % and 20 % of that of **1**, with an overall higher production by the W160A in comparison to the F86A variant. Gratifyingly, the C59A variant gave a similar product profile, but with a much better production (about 6‐fold in comparison to the F86A variant except for **3**). Exchanges of other active site residues (C82A, A229G and N315A) led to no significant product change compared to wild‐type CyS (Figure S3).

Compounds **2**–**6** were isolated from large scale fermentations of engineered *E. coli* expressing the CyS A190G or C59A variety, and their structures were elucidated by NMR spectroscopy. All five diterpenes exhibit different carbon skeletons (Figure 1B) and are formed from several of the proposed pathway intermediates. Compound **2** arises by deprotonation at C‐6 of **G**, **3** is generated through proton abstraction at C‐20 of **D**, and allokutznerene^[16]^ (**4**) and **5** originate from alternative deprotonations of **C** (Figure 3 and S6). The production of **3**–**6** from the enzyme variants of F86A and W160A supports the role of these residues in stabilizing intermediates **D** and **E**. The F86A and W160A variants are incompetent to stabilize these intermediates which consequently leads to shunt products derived from **C** and **D** (Figure 3).

For a deeper understanding of the increased but less selective production by the C59A variant (Cys^C59A^), its crystal structure was solved at 2.30 Å. The structure is highly similar to the apo‐CyS and CyS‐GGPP‐Mg^2+^ structures (Figure 5A, RMSD of 0.16 Å and 0.15 Å for Cα atoms). The active site residues of the three structures superimpose well, with the exception of F86 located between C59 and the substrate binding pocket (Figure 5B). In the apo‐CyS and CyS‐GGPP‐Mg^2+^ structures, the thiol of C59 is close to the phenyl ring of F86 (3.4 Å) and renders it towards the active site, while in CyS^C59A^ this interaction is disrupted. As a result, F86 moves a bit away from the active site and the phenyl ring rotates 24° clockwise (Figure 5B). This slightly widens the active site cavity which may lead to an improved uptake of GGPP, albeit on the expense of selectivity because of a less tight substrate control through cation‐π interactions. Similar observations have been made before for SvS,^[22]^ which together with our results provides a basis for future TS engineering strategies.

**Figure 5:**
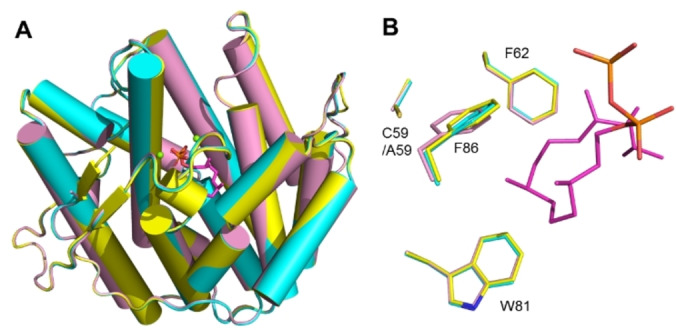
Comparison of CyS^C59A^ with apo‐CyS and CyS‐GGPP‐Mg^2+^. A) Superimposition of CyS^C59A^ (pink) with apo‐CyS (cyan) and CyS‐GGPP‐Mg^2+^ (yellow). B) Comparison of Phe86 in apo‐CyS, CyS‐GGPP‐Mg^2+^ and CyS^C59A^. GGPP is shown as magenta sticks.

Compound **6** possesses a novel skeleton and the mechanism of its formation was further investigated. For this purpose, all 20 isotopomers of (^13^C)GGPP, prepared enzymatically from ^13^C‐labeled FPP, GPP or IPP precursors (Table S8), were enzymatically converted with CyS^C59A^, followed by extraction with C_6_D_6_ and analysis of the product mixture through ^13^C NMR (Figure S34 and S35). All 20 experiments resulted in the detection of the labeled carbons of the six products, with one signal matching the NMR data of **6** in each experiment. The results revealed a remarkable mechanism for its formation with the first steps towards **C** being the same as for the other products, but then branching out through **I** to **O** with involvement of multiple ring closures, 1,2‐hydride shifts, and skeletal rearrangements (Figure 6). The 1,2‐hydride shift from intermediate **I** to **J** was investigated with (3‐^13^C,2‐^2^H)FPP^[30]^ and IPP with GGPP synthase (GGPPS)^[13]^ and CyS^C59A^, resulting in a slightly upfield shifted triplet for C‐7 of **6** (Figure S36A and B) due to a direct ^13^C‐^2^H bond in the product. The 1,2‐hydride shifts from **K** to **L** and from **N** to **O** represent a forward and backward movement of the same hydrogen. Consequently, when using (3‐^13^C,2‐^2^H)GGPP^[13]^ with CyS^C59A^ the deuterium atom will end up in its starting position, in agreement with the observed minor upfield shift for the signal of C‐3 of **6**, indicating a deuterium atom in a neighbouring position (Figure S36C and D). The stereoselectivity of the final deprotonation was investigated by conversion of DMAPP and (*E*)‐ or (*Z*)‐(4‐^2^H, 4‐^13^C)IPP^[16]^ with GGPPS and CyS^C59A^, showing loss of deuterium from (*E*)‐(4‐^2^H,4‐^13^C)IPP and retainment from (*Z*)‐(4‐^2^H,4‐^13^C)IPP, i.e. loss of the α‐oriented proton in **O** (Figure S37).

**Figure 6:**
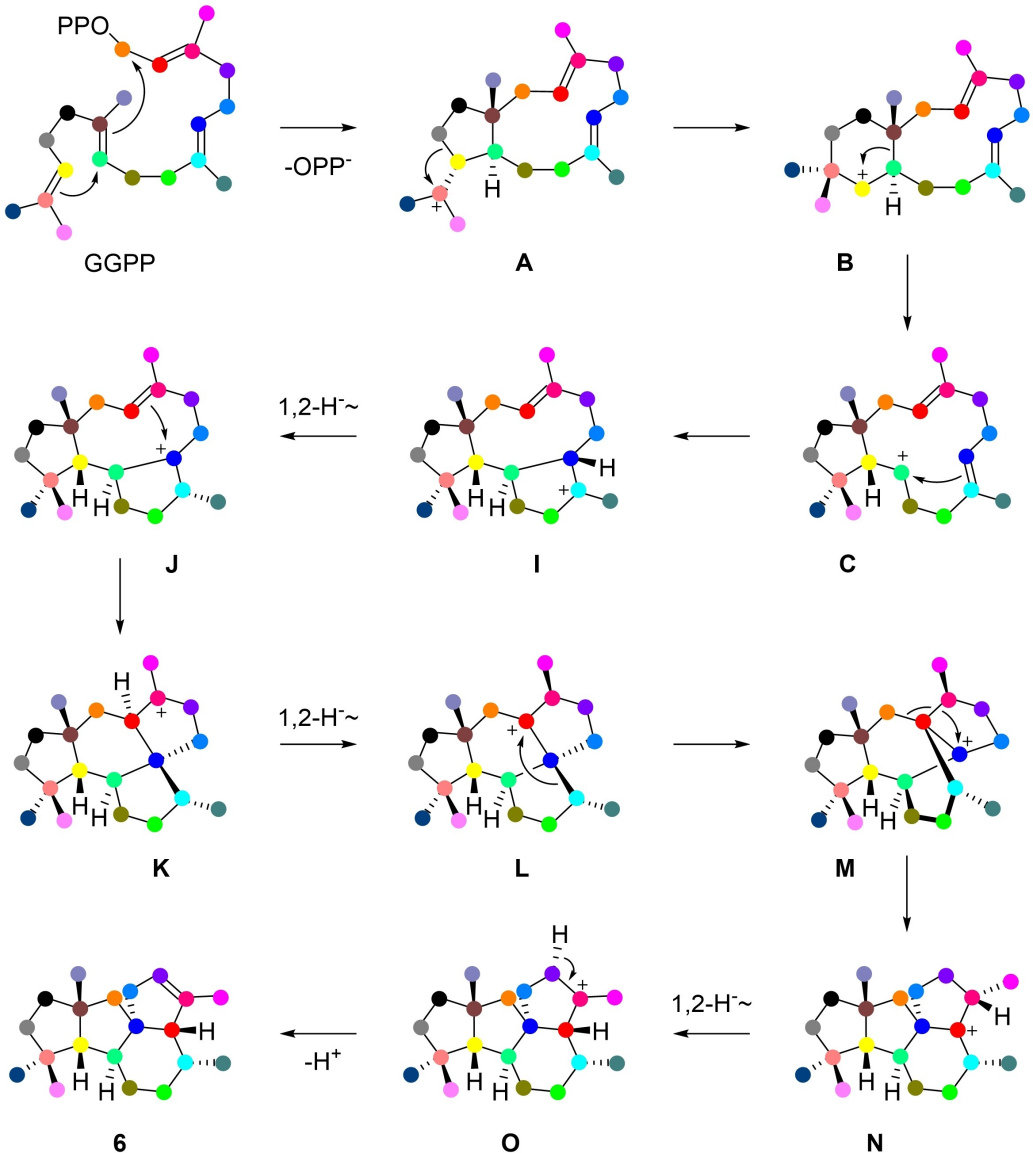
Cyclization mechanism from GGPP to **6**.

In summary, the structures of CyS and CyS‐GGPP‐Mg^2+^, representing the first example of a TS in complex with its native substrate, were solved. Intermediate modelling and site‐directed mutagenesis gave detailed insights into the biosynthesis of **1**. Based on the structure, several CyS variants were designed, and especially CyS^C59A^ showed a strongly altered product profile with formation of several new compounds. The C59A exchange widens the active site cavity, resulting in a relaxed product selectivity, with a series of unprecedented ring closures and skeletal rearrangements towards **6**. Previous site‐directed mutageneses revealed critical TS residues for functionality, while exchanges of other residues lead to changed product profiles.^[31]^ Amino acid sequence alignments to selinadiene synthase, for which the structure and active site residues are known, allow for an identification of residues that presumably contour the active site cavities of other enzymes. This enabled the generation of enzyme variants of polytrichastrene synthase to obtain novel products.^[20]^ However, such alignment based targetings cannot fully substitute for the structure based identification of residues, and in fact the position analogous to C59 in CyS has not been targeted in any other TS before. Even more structural information will be required to deepen our understanding of TS catalysis and to open the possibility of sequence‐function predictions and rational enzyme engineering.

## Conflict of interest

The authors declare no conflict of interest.

## Supporting information

As a service to our authors and readers, this journal provides supporting information supplied by the authors. Such materials are peer reviewed and may be re‐organized for online delivery, but are not copy‐edited or typeset. Technical support issues arising from supporting information (other than missing files) should be addressed to the authors.

Supporting Information

## Data Availability

The data that support the findings of this study are available in the Supporting Information of this article. Crystal structures have been deposited in the Protein Data Bank with PDB IDs 7Y50 (apo‐CyS), 7Y88 (CyS‐GGPP‐Mg^2+^), and 7Y87 (CyS^C59A^).
