# Supplementary material for: Crystal Structure Based Mutagenesis of Cattleyene Synthase Leads to the Generation of Rearranged Polycyclic Diterpenes
Source: Angew Chem Int Ed Engl. 2022 Aug 1;61(36):e202209785. doi: 10.1002/anie.202209785 (PMC9543850; doi:10.1002/anie.202209785)
Supplement: Supplementary file 1 — Supporting Information [file ANIE-61-0-s001.pdf]

Angewandte Chemie

Eine Zeitschrift der Gesellschaft Deutscher Chemiker

Supporting Information

Crystal Structure Based Mutagenesis of Cattleyene Synthase Leads to the Generation of Rearranged Polycyclic Diterpenes

B. Xing, H. Xu, A. Li, T. Lou, M. Xu, K. Wang, Z. Xu, J. S. Dickschat, D. Yang*, M. Ma**

Table of Contents

1. General experimental procedures ··· S4
2. Gene expression and protein purification of CyS ··· S4
3. Crystallization, data collection and structural elucidation of CyS and CyS-GGPP-Mg^{2+} complex S4
4. Crystallization, data collection and structural elucidation of CyS^{C59A} ··· S5
5. Substrate modelling ··· S5
6. Construction of engineered *E. coli* heterologous systems ··· S5
7. Site-directed mutagenesis ··· S6
8. The GC-MS analysis and isolation of diterpenes ··· S6
9. Structural elucidation of diterpenes ··· S6
10. Isotopic labeling experiments ··· S7
Table S1. Primers for plasmids construction in this study ··· S9
Table S2. Plasmids and strains used in this study ··· S11
Table S3. The information of synthesized genes in the construction of engineered *E. coli* strains ··· S13
Table S4. The data collection and refinement statistics of crystal structures ··· S14
Table S5. The ^1H NMR data of **1-6** in $CDCl_3$ ··· S15
Table S6. The ^{13}C NMR data of **1-6** in $CDCl_3$ ··· S17
Table S7. Top 10 homologues of CyS from the DALI alignment ··· S18
Table S8. Isotopic labeling experiments with CyS^{C59A} ··· S19
Figure S1. The SDS-PAGE and Mg^{2+}-binding network ··· S20
Figure S2. The construction of engineered *E. coli* strains ··· S21
Figure S3. GC-MS analysis of the products of CyS mutants ··· S22
Figure S4. Mass spectra of compounds **1-6** identified from GC-MS analysis ··· S23
Figure S5. The key 2D NMR correlations of new diterpene variants ··· S24
Figure S6. The proposed biosynthetic pathways of **2-5** ··· S25
Figure S7. The ^1H NMR and ^{13}C NMR spectrum of compound **1** in $CDCl_3$ ··· S26
Figure S8. The ^1H NMR and ^{13}C NMR spectrum of compound **2** in $CDCl_3$ ··· S27
Figure S9. The COSY spectrum of compound **2** in $CDCl_3$ ··· S28
Figure S10. The HSQC spectrum of compound **2** in $CDCl_3$ ··· S28
Figure S11. The HMBC spectrum of compound **2** in $CDCl_3$ ··· S29
Figure S12. The DEPT 135° spectrum of compound **2** in $CDCl_3$ ··· S29
Figure S13. The NOESY spectrum of compound **2** in $CDCl_3$ ··· S30
Figure S14. The IR spectrum of compound **2** ··· S30
Figure S15. The HRMS (EI) spectrum of compound **2** ··· S31
Figure S16. The ^1H NMR and ^{13}C NMR spectrum of compound **3** in $CDCl_3$ ··· S32
Figure S17. The ^1H NMR and ^{13}C NMR spectrum of compound **4** in $CDCl_3$ ··· S33
Figure S18. The ^1H NMR and ^{13}C NMR spectrum of compound **5** in $CDCl_3$ ··· S34
Figure S19. The COSY spectrum of compound **5** in $CDCl_3$ ··· S35
Figure S20. The HSQC spectrum of compound **5** in $CDCl_3$ ··· S35
Figure S21. The HMBC spectrum of compound **5** in $CDCl_3$ ··· S36
Figure S22. The DEPT 135° spectrum of compound **5** in $CDCl_3$ ··· S36
Figure S23. The NOESY spectrum of compound **5** in $CDCl_3$ ··· S37
Figure S24. The IR spectrum of compound **5** ··· S37
Figure S25. The HRMS (EI) spectrum of compound **5** ··· S38

Figure S26. The ^{1}H NMR and ^{13}C NMR spectrum of compound **6** in $CDCl_3$ ·························S39
Figure S27. The COSY spectrum of compound **6** in $CDCl_3$ ·······································S40
Figure S28. The HSQC spectrum of compound **6** in $CDCl_3$ ·······································S40
Figure S29. The HMBC spectrum of compound **6** in $CDCl_3$ ·······································S41
Figure S30. The DEPT 135° spectrum of compound **6** in $CDCl_3$ ·································S41
Figure S31. The NOESY spectrum of compound **6** in $CDCl_3$ ·····································S42
Figure S32. The IR spectrum of compound **6** ···S42
Figure S33. The HRMS (EI) spectrum of compound **6** ···S43
Figure S34. ^{13}C-NMR spectra of (^{13}C)GGPP labeled at carbons C-1 – C-10with CyS^{C59A} ···········S44
Figure S35. ^{13}C-NMR spectra of (^{13}C)GGPP labeled at carbons C-11 – C-20 with CyS^{C59A} ·········S45
Figure S36. 1,2-Hydride shifts in the biosynthesis of **6** ··S46
Figure S37. The stereochemical course of the deprotonation to **6** ·································S47
References ···S48

1. General experimental procedures

Optical rotations were measured on an Autopol III automatic polarimeter (Rudolph Research Analytical) equipped with a 0.2 mL cuvette. IR spectra were recorded with a NICOLET iS50 FT-IR spectrometer (Thermo Scientific, Waltham, MA, USA). NMR data were collected on Bruker Avance-600 and Avance-700 NMR spectrometers (Bruker Corporation, Billerica, MA, USA). GC-MS analyses were performed on an Agilent 7890A GC system connected to an Agilent 5975 mass spectrometer with a HP-5MS column (30 m ×250 μm × 0.25 μm, Agilent Technologies, Santa Clara, CA, USA). HPLC analysis was performed on an Agilent 1260 series (Agilent Technologies, Santa Clara, CA, USA) with a C_{18} RP-column (Extend-C_{18}, 250 × 4.6 mm, 5 μm, Agilent Technologies, Santa Clara, CA, USA). Semi-preparative HPLC was performed on an SSI 23201 system (Scientific Systems Inc., State College, PA, USA) with a YMC-Pack ODS-A column (250 × 10 mm, 5 μm, YMC CO., LTD. Shimogyo-ku, Kyoto, Japan). All fermentations were carried out in MQD-B1R shakers (Minquan Instrument Co., Ltd., Shanghai, China).

2. Gene expression and protein purification of CyS

The nucleotide sequence encoding the CyS enzyme was codon-optimized and synthesized for expression in *E. coli*. The DNA fragment of *cyS* with 15-20 bp homologous arms was amplified by PCR with primer cyS-pET28a-F and cyS-pET28a-R (Table S1). The purified PCR product of *cyS* was fused to a C-terminal hexahistidine tag in pET28a vector through Gibson Assembly method and transformed into *E. coli* BL21 Fast-T1 (Vazyme Biotech Co., Ltd., Nanjing, China). The resulting plasmid was confirmed by sequencing.

The plasmid was transformed into *E. coli* BL21 (DE3) (TransGen Biotech, Beijing, China). Overexpression was performed using lysogeny broth (LB) medium with 30 μg/mL kanamycin at 37 °C, 220 rpm until an OD_{600} of 0.6 was reached, then medium was subsequently cooled to 18 °C with the addition of isopropyl β-D-1-thiogalactopyranoside (IPTG, final concentration 0.20 mM) for 18 h.

The cells were harvested by centrifugation at 4000 rpm for 10 min at 4 °C. The pellet was resuspended in lysis buffer (100 mM Tris, pH 8.0, containing 300 mM NaCl, 15 mM imidazole, and 10% glycerol), lysed by sonication, and centrifuged at 10000 rpm for 30 min at 4 °C. The supernatant containing CyS was filtered by 0.45 μm membrane and loaded onto a 5 mL Histrap HP column (GE Healthcare Life Sciences) with the ÄKTA FPLC system, then CyS proteins were eluted with buffer A (50 mM Tris, 100 mM imidazole, and 300 mM NaCl, pH 8.0). The fractions containing CyS proteins were exchanged to buffer B (20 mM Tris and 10 mM NaCl, pH 8.0) using PD10 column (GE Healthcare Life Sciences) and loaded onto an anion-exchange Resource Q column (GE Healthcare Life Sciences), which was eluted with an increased gradient of buffer B to buffer C (20 mM Tris and 1 M NaCl, pH 8.0). Purified proteins were analyzed by SDS-PAGE and the concentration was determined at 280 nm with a NanoDrop 2000C spectrophotometer (Thermo Scientific, Waltham, MA, USA). The homogeneity of CyS was analyzed by SDS-PAGE and shown in Figure S1.

3. Crystallization, data collection and structural elucidation of CyS and CyS-GGPP-Mg^{2+} complex

The crystallization screening of CyS (20 mg/mL) was performed in 24-well plates with the hanging-drop vapor-diffusion method at 16 °C, and crystals of CyS were observed after 3 days in a screening kit Classic 1-14 (Rigaku) that contains 1000 mM Sodium citrate tribasic and 100 mM Sodium cacodylate / Hydrochloric acid pH 6.5. To obtain complex crystals of CyS with GGPP, CyS crystals were soaked in Classic 1-14 containing 2.0 mM GGPP and 2.5 mM $MgCl_2$ for 24 h at 16 °C.

The X-ray diffraction data of apo-CyS and CyS-GGPP-Mg^{2+} complex were collected at beamline BL02U1 at the Shanghai Synchrotron Radiation Facility (SSRF) with a wavelength of 0.97918 Å.[1] The diffraction data were processed and scaled with HKL-2000.[2] The crystal structure of apo-CyS was solved by molecular replacement using SvS as the searching model.[3] The initial phases of the CyS-GGPP-Mg^{2+} complex structure was determined by molecular replacement, using the apo structure of CyS as a search model. Molecular replacement was performed with Phaser in PHENIX.[4,5] The structure was modified manually with Coot[6] and refined with PHENIX[7].

4. Crystallization, data collection and structural elucidation of CyS^{C59A}

The crystallization screening of CyS^{C59A} (20 mg/mL) was performed in 24-well plates with the hanging-drop vapor-diffusion method at 16 °C, and crystals of CyS^{C59A} were observed after 2 days in a screening kit Index 18 (Hampton Research) that contains 0.49 M Sodium phosphate monobasic monohydrate, 0.91 M Potassium phosphate dibasic, pH 6.9.

The X-ray diffraction data of CyS^{C59A} was collected at at 100 K in a liquid nitrogen stream, using a HyPix 6000HE detector at XtaLAB Synergy with a wavelength of 1.54184 Å. The data sets were processed and scaled using *CrysAlis^{Pro}* software.[8] The crystal structure of CyS^{C59A} was solved by molecular replacement with Phaser using apo-CyS structure as the searching model. Molecular replacement was performed with Phaser in PHENIX. The structure was modified manually with Coot and refined with PHENIX.

5. Substrate modelling

Substrate modeling was performed using AutoDock Vina 1. 1. 2.[9] Before the docking, water molecules were removed from complex structure CyS-GGPP-Mg^{2+} and manually remove the hydrocarbon chain of GGPP. AutoDockTools 1.5.6 was used to prepare the intermediates **A**-**H**, macromolecules CyS-GGPP-Mg^{2+} complex, and grid map files. The default parameters were used to set the torsion constraints for intermediates **A**-**H**, and charges and hydrogen atoms were added to CyS-GGPP-Mg^{2+} complex proteins. For the parameters of CyS-GGPP-Mg^{2+} complex with intermediates **A**-**H**, a grid box of 12.75 × 10.50 × 15.0 nm^3 (x × y × z) with the search space center of x = 50.745, y = 28.426, and z = 51.073 was created. The search parameters were used with default values.

6. Construction of engineered *E. coli* heterologous systems

To improve the production of diterpenes, we reconstructed isopentenol utilization pathway (IUP) which can produce isopentenyl diphosphate (IPP) and dimethylallyl diphosphate (DMAPP) in *E. coli* (Figure S2).[10] The biosynthetic genes *ecTHIM*, *mjIPK*[11] and *idi*[12] were amplified by PCR and individually cloned into plasmid pCDFDuet-1 with the Gibson assembly method to produce IPP and DMAPP, and the GGPP synthase-encoding gene *ptmT4* (Table S3) and *cyS* were cloned into plasmid pRSFDuet-1 with the same method.

The two plasmids pCDFDuet-*ecTHIM-mjIPK-idi* and pRSFDuet-*ptmT4-cyS* were co-transformed into *E. coli* BL21 (DE3). The positive transformants were cultivated in 50 mL LB supplemented with 30 μg/mL kanamycin and 50 μg/mL spectinomycin at 37 °C, 220 rpm for 8 h to afford seed cultures. The seed culture was inoculated into TB medium (tryptone 12 g, yeast extract 24 g, glycerol 4 mL, KH_2PO_4 2.31 g, K_2HPO_4 12.54 g in 1 L distilled H_2O) with 30 μg/mL kanamycin and 50 μg/mL spectinomycin. The fermentation was continued at 37 °C, 220 rpm until an OD_{600} of 0.8 was reached, then the culture was cooled down to 18 °C. At 18 °C, IPTG (final concentration 0.5 mM) was added for induction and

isoprenol (final concentration 25 mM) was fed at the same time. The fermentation was continued for additional 72 h at 18 °C and analyzed by GC-MS.

7. Site-directed mutagenesis

The site-directed mutagenesis experiments of *cyS* were carried out by PCR using two overlapping primers (Table S1) containing the mutated sequences with pRSFDuet-*ptmT4-cyS* as the template, to generate a series of mutated plasmids. Subsequently, the parental vector is digested by *Dpn*I and the mixture is transformed into *E. coli* Fast-T1. The resulting plasmids were confirmed by sequencing. These mutant plasmids were individually co-transformed into *E. coli* with pCDFDuet-*ecTHIM-mjIPK-idi* plasmid for subsequent fermentation and detection.

8. The GC-MS analysis and isolation of diterpenes

The fermentation broths were extracted with an equal volume of hexane, and the hexane extracts were dried under vacuum with a rotary evaporator. For GC-MS analysis, a small part of the hexane extracts was dissolved in hexane and subjected to GC-MS analysis. Samples (1 μL) were injected in splitless mode at 50 ℃. After being held at 50 ℃ for 3 min, the oven temperature was raised to 300 ℃ with a rate of 14 ℃/min, then held for another 3 min.

For isolation of diterpenes, large-scale fermentations were carried out as the same procedures above. The crude extract was subjected to silica gel flash chromatography eluted with hexane to yield a series of fractions. Fractions containing diterpenes were monitored by TLC and purified by Semi-preparative HPLC eluted with 100% acetonitrile (0 - 60 min) at a flow rate of 2.0 mL/min, under the UV detection at 210 nm.

9. Structural elucidation of diterpenes

Compound **2** was obtained as colorless oil. $[\alpha]^{25}_{D}$ +48.0 (*c* 0.05, hexane). IR (diamond ATR) ν_{max}: 3385, 2931, 2864, 1707, 1461, 1373 cm^{-1}. HREIMS analysis afforded an M^+ ion at *m/z* 272.24963 (calcd for $C_{20}H_{32}$, 272.24985), giving the molecular formula of **2** as $C_{20}H_{32}$. The ^{1}H NMR spectrum of **2** resembled that of **1** except that the resonance at δ_H 1.92 (s, H-1) in **1** is shielded to δ_H 1.57 (br s, H-1) in **2**, while the resonance at δ_H 1.66 (m, H-8a) and 1.13 (m, H-8b) in **1** is deshielded to δ_H 2.20 (m, H-8a) and 2.15 (m, H-8b) in **2**; the resonance at δ_H 0.89 (d, J = 6.5 Hz, H-19) in **1** is replaced by δ_H 1.57 (s, H-19) in **2**, and the resonance at δ_H 1.39 (m, H-6) in **1** is replaced by δ_H 2.00 (br q, H-2) in **2** (Table S5). Compared to the ^{13}C NMR spectrum of **1**, the ^{13}C NMR spectrum of **2** shows significantly changed resonances towards those attributed to C-1–C-8 and C-20 in **1** (Table S6). Those differences above suggest that **2** contains a double bond at C-6/C-7 instead of C-2/C-3 in **1**, and contains a methyl group (C-20) attached on C-3 instead of C-7. The COSY, HSQC, and HMBC correlations of **2** confirm the different positions of double bond and C-20 methyl group between **1** and **2** (Figure S5). Since **2** is produced from later cyclization intermediate **G** in the biosynthesis of **1** (Figure 3), we deduce that the absolute configurations of C-11, C-14, and C-10 in **2** are consistent with those in **1**. The NOESY spectrum of **2** shows the correlations from H-10 to H-2, suggesting that H-10 and H-2 are on the same side of the ring and establishing the absolute configuration of C-2 as *R*. The NOESY correlations from H-20 to H-18 and H-4a, H-2 to H-4b confirm the *S* configuration at C-3 in **2**. Thus, compound **2** is established as a new diterpene with similar 5/5/6/5 ring system as **1**.

Compound **3** was identified as a variediene analogue that has been reported as the product of a promiscuous terpene synthase FgJ07623,[13] Compound **4** was identified as allokutznerene that has been

reported as the product of a diterpene synthase PmS from *Allokutzneria albata*.[14]

Compound **5** was obtained as colorless oil. $[\alpha]^{25}_{D}$ +59.3 (*c* 0.10, hexane). IR (diamond ATR) ν_{max}: 2946, 2867, 1454, 1375 cm^{-1}. HREIMS analysis afforded an M^+ ion at *m/z* 272.24963 (calcd for $C_{20}H_{32}$, 272.24985), giving the molecular formula of **5** as $C_{20}H_{32}$. The ^1H NMR spectrum of **5** resembled that of **4** except that the resonance at δ_H 2.21 (d, J = 16.0 Hz, H-1a) and 1.76 (m, H-1b) in **4** are replaced by δ_H 5.39 (s, H-1) in **5**; a new resonance at δ_H 2.49 (m, H-3) occurs in **5**, and the resonance at δ_H 1.65 (s, H-20) in **4** is replaced by δ_H 1.08 (d, J = 7.1 Hz, H-20) in **5**; and the resonance at δ_H 2.34 (m, H-4a) and 2.07 (m, H-4b) in **4** is shielded to δ_H 1.82 (m, H-4a) and 1.18 (m, H-4b) in **5** (Table S5). Compared to the ^{13}C NMR spectrum of **4**, the ^{13}C NMR spectrum of **5** shows different double bond chemical shifts (Table S6). Those differences above suggest that **5** only differs from **4** by containing a double bond at C-1/C-2 instead of C-2/C-3 in **4**, which is confirmed by the COSY, HSQC, and HMBC correlations of **5** (Figure S5). Since **5** shares the same tetracyclic ring formation pathway as **4** and only differs from **4** in the later carbocation intermediates and proton abstraction positions (Figure S6), we deduce that the absolute configurations of C-11, C-14, C-10, and C-6 in **5** are the same as those in **4**. The NOESY spectrum of **5** shows the correlations from H-19 to H-20, H-14 and H-18, suggesting that H-20 and H-19 are on the same side of the ring and establishing the absolute configuration of C-3 as *S*. Thus, compound **5** is established as a new diterpene with distinct tetracyclic ring system from **1**.

Compound **6** was obtained as colorless oil. $[\alpha]^{25}_{D}$ +37.5 (*c* 0.08, hexane). IR (diamond ATR) ν_{max}: 2928, 2869, 1453, 1371 cm^{-1}. HREIMS analysis afforded an M^+ ion at *m/z* 272.24960 (calcd for $C_{20}H_{32}$, 272.24985), giving the molecular formula of **6** as $C_{20}H_{32}$. The ^1H and ^{13}C NMR spectra of **6** show similar chemical shifts and coupling constants to those of the substituted 5/5 bicyclic ring system in **1** (Table S5 and S6). The COSY, HSQC, and HMBC spectra of **6** confirm the presence of the substituted 5/5 bicyclic ring in **6** (Figure S5). The presence of a six-membered ring fused to the 5/5 ring is confirmed by a continuous COSY correlation system beginning from the 5/5 ring (H-14, H-10, H-9, H-8, H-7, and H-3), and the HMBC correlations from H-3 to C-1, C-2 and C-10 (Figure S5). One methyl group (C-19) attached on C-7 is confirmed by the COSY correlation from H-7 to H-19, and the HMBC correlations from H-19 to C-3 and C-8 (Figure S5). The COSY correlation from H-5 to H-6, in combination with the HMBC correlations from H-5 to C-4 and C-20, H-20 to C-4 and C-5, H-6 to C-5 and C-4, established the presence of a 1,3-disubstituted 2-butylene moiety. The attachment of the 1,3-disubstituted 2-butylene moiety on C-2 and C-3 is identified by the HMBC correlations from H-5 to C-2, H-6 to C-2, H-20 to C-3, H-5 to C-3 (Figure S5). Thus, the planar structure of **6** is established as a distinct 5/5/6/5 tetracyclic ring system from those of **1**–**5**. Compound **6** shares the same biosynthetic pathway to **1** towards the first five-membered ring formation (Figure 6), therefore the absolute configurations of C-11 and C-14 in **6** are deduced as the same to those in **1**. The NOESY correlations from H-10 to H-16, H-14 to H-17 suggest that H-10 and H-14 are on the different sides of the ring and the absolute configuration of C-10 is *S* (Figure S5). The NOESY correlation from H-10 to H-6 shows that they are on the same side of the ring and the absolute configuration of C-2 is *R*; the NOESY correlations from H-3 to H-18 and H-1 establish the absolute configuration of C-3 is *S*; and the NOESY correlations from H-19 to H-20 establish the absolute configuration of C-7 is *R* (Figure S5). Thus, compound **6** is identified as a new diterpene with a novel carbon skeleton.

10. Isotopic labeling experiments

The isotopic labeling experiments were performed with the substrates and enzymes as listed in Table S8. The reaction mixtures contained substrates (1 mg each) in aqueous NH_4HCO_3 solution (1 mL, 25 mM),

enzyme elution fractions (1 mL each) and incubation buffer (5 mL, 50 mM Tris, 10 mM $MgCl_2$, 20 vol-% glycerol, pH 8.2). After incubation with shaking at 28 °C overnight, the reaction mixtures were extracted with C_6D_6 (0.6 mL + 0.2 mL). The extracts were dried with $MgSO_4$ and analysed by GC-MS and NMR.

Table S1. Primers for plasmids construction in this study

Primers	Sequence (5' to 3')
Cys-pET28a-F	GTGGTGCTCAGTTGCAGGGCGACCACCCAGCGGATC
Cys-pET28a-R	GAGATATACCATGGGCATGCCGGTTCCGGGCACCC
pET28a-F	GCCCATGGTATATCTCCTTCTTAAAG
pET28a-R	TCAACTGAGCACCACCACCAC
EcTHIM-pCDFDuet-F	GAGATATACCATGGATGCAGGTAGATTTACTATCAAGTGCTC
EcTHIM-pCDFDuet-R	CTCGAATTCGGATCCTTATGCTTGCACTTCTTGGGTCAG
pCDFDuet-EcTHIM-F	GGATCCGAATTCGAGCTC
pCDFDuet-EcTHIM-R	CCATGGTATATCTCCTTATTAAAGTTAAAC
MjIPK-pCDFDuet-F	AGGAGATATACATATGATGTTAACAATATTGAAACTAGGAGGGTC
MjIPK-pCDFDuet-R	CTCGAGGGTACCTTATTCGCTAAAATCAATCTCCGTACC
pCDFDuet-MjIPK-F	GGTACCCTCGAGTCTGGTAAAGAAACCGC
pCDFDuet-MjIPK-R	CATATGTATATCTCCTTCTTATACTTAACTAATATACTAAGATGGG
idi-pCDFDuet-F	TAAGGTACCCTCGAGATGCAAACAGAACACGTAATACTATTAAA
idi-pCDFDuet-R	GCCTAGGTTAATTAATTACTTCAACTGGGTGAACGCAGAC
pCDFDuet-idi-F	TTAATTAACCTAGGCTGCTGCCAC
pCDFDuet-idi-R	CTCGAGGGTACCTTATTCGCTAAAATCAATC
PtmT4-pRSFDuet-F	GGAGATATACATATGGTTCACGCTGACACCGTTCAG
PtmT4-pRSFDuet-R	CTTTACCAGACTCGAGTTAGTGTTTACGGAAAGCAACGTAGTC
pRSFDuet-PtmT4-F	CTCGAGTCTGGTAAAGAAACCGCTG
pRSFDuet-PtmT4-R	CATATGTATATCTCCTTCTTATACTTAACTAATATACTAAGATGGGG
CyS-pRSFDuet-F	GAAGGAGATATACATATGATGCCGGTTCCGGGCACCC
CyS-pRSFDuet-R	CAGACTCGAGGGTACCTTAAGGGCGACCACCCAGCGGATCC
PRSFDuet-CyS-F	GGTACCCTCGAGTCTGGTAAAGAAAC
PRSFDuet- CyS-R	CATATGTATATCTCCTTCTTATACTTAACTAATATACTAAG
CyS-C59A-F	GAATGCGTGGTAATGATGCACCGGGCTTTTATGG
CyS-C59A-R	GTGCATCATTACCACGCATTCGCGCACGCTGC
CyS-F62A-F	GGTAATGATTGTCCGGGCGCGTATGGCCGCATTATG
CyS-F62A-R	AGCAGCACGGTTAGCAACACGCAGCTGTTC
CyS-W81A-F	GCAGTTAGCGGTGGATGCGTGCACCGTGATG
CyS-W81A-R	CGCATCCACCGCTAACTGCAGGCGATCGGTC
CyS-C82A-F	GTTAGCGGTGGATTGGGCGACCGTGATGTTTC
CyS-C82A-R	TCGCCCAATCCACCGCTAACTGCAGGCGATC
CyS-F86A-F	TTGGTGCACCGTGATGGCGCATTTTGATGATG
CyS-F86A-R	GCGCCATCACGGTGCACCAATCCACCGCTAAC
CyS-W160A-F	GAAGCGCATCGTGCGGCGTTTTTAGCGGTGG
CyS-W160A-R	ACGCCGCACGATGCGCTTCCGCACAACGACG
CyS-A190G-F	TATGCGCCAGCATACCGGCGCGGGCGCAGCAAC
CyS-A190G-R	CGCCGGTATGCTGGCGCATATGCGCATAATCAT
CyS-A191G-F	GCGCCAGCATACCGCGGGCGGCGCAGCAACT
CyS-A191G-R	CGCCCGCGGTATGCTGGCGCATATGCGCATAATC
CyS-A229G-F	GCGTTTACCACCGCGGGCTTTGATGATGATC

CyS-A229G-R	AGCCCGCGGTGGTAAACGCTAATTCGGTTAACG
CyS-N315A-F	CATCTGCTGCCGGGCGCGCTGGAATGGGGCTTAAC
CyS-N315A-R	CGCGCCCGGCAGCAGATGGCACAGATGATCTA
CyS-W318A-F	CCGGGCAACCTGGAAGCGGGCTTAACCGC
CyS-W318A-R	CCCGCTTCCAGGTTGCCCGGCAGCAGATGG

Table S2. Plasmids and strains used in this study.

Name	Description	Source
**Plasmids**		
pET28a (+)	Gene expression plasmid used in *E. coli*, encoding N-terminal His_6 tag, kanamycin resistance	Beijing Tiandz Biotech Co., Ltd
pCDFDuet-1	Gene expression plasmid used in *E. coli*, containing two multiple cloning sites; kanamycin resistance	Novagen
pRSFDuet-1	Gene expression plasmid used in *E. coli*, containing two multiple cloning sites; streptomycin resistance	Novagen
pMM3001	pET-28a (+)-derived plasmid bearing the *cyS* gene	This study
pMM3002	pCDFDuet-1-derived plasmid bearing the *ecTHIM*, *mjIPK*, *idi* gene	This study
pMM3003	pRSFDuet-1-derived plasmid bearing the *ptmT4*, *cyS* gene	This study
pMM3004	pRSFDuet-1-derived plasmid bearing the *ptmT4*, *cyS-C59A* gene	This study
pMM3005	pRSFDuet-1-derived plasmid bearing the *ptmT4*, *cyS-F62A* gene	This study
pMM3006	pRSFDuet-1-derived plasmid bearing the *ptmT4*, *cyS-W81A* gene	This study
pMM3007	pRSFDuet-1-derived plasmid bearing the *ptmT4*, *cyS-C82A* gene	This study
pMM3008	pRSFDuet-1-derived plasmid bearing the *ptmT4*, *cyS-F86A* gene	This study
pMM3009	pRSFDuet-1-derived plasmid bearing the *ptmT4*, *cyS-W160A* gene	This study
pMM3010	pRSFDuet-1-derived plasmid bearing the *ptmT4*, *cyS-A190G* gene	This study
pMM3011	pRSFDuet-1-derived plasmid bearing the *ptmT4*, *cyS-A191G* gene	This study
pMM3012	pRSFDuet-1-derived plasmid bearing the *ptmT4*, *cyS-A229G* gene	This study
pMM3013	pRSFDuet-1-derived plasmid bearing the *ptmT4*, *cyS-N315A* gene	This study
pMM3014	pRSFDuet-1-derived plasmid bearing the *ptmT4*, *cyS-W318A* gene	This study
pMM3015	pET-28a (+)-derived plasmid bearing the *cyS-C59A* gene	
***E. coli* strains**		
E. coli BL21(DE3)	Heterologous host for protein production	Beijing TransGen Biotech Co., Ltd

MM30001	*E. coli* BL21(DE3) with the plasmid pMM3001 transformed	This study
MM30002	*E. coli* BL21(DE3) with the plasmid pMM3002 and pMM3003 transformed	This study
MM30003	*E. coli* BL21(DE3) with the plasmid pMM3002 and pMM3004 transformed	This study
MM30004	*E. coli* BL21(DE3) with the plasmid pMM3002 and pMM3005 transformed	This study
MM30005	*E. coli* BL21(DE3) with the plasmid pMM3002 and pMM3006 transformed	This study
MM30006	*E. coli* BL21(DE3) with the plasmid pMM3002 and pMM3007 transformed	This study
MM30007	*E. coli* BL21(DE3) with the plasmid pMM3002 and pMM3008 transformed	This study
MM30008	*E. coli* BL21(DE3) with the plasmid pMM3002 and pMM3009 transformed	This study
MM30009	*E. coli* BL21(DE3) with the plasmid pMM3002 and pMM3010 transformed	This study
MM30010	*E. coli* BL21(DE3) with the plasmid pMM3002 and pMM3011 transformed	This study
MM30011	*E. coli* BL21(DE3) with the plasmid pMM3002 and pMM3012 transformed	This study
MM30012	*E. coli* BL21(DE3) with the plasmid pMM3002 and pMM3013 transformed	This study
MM30013	*E. coli* BL21(DE3) with the plasmid pMM3002 and pMM3014 transformed	This study
MM30014	*E. coli* BL21(DE3) with the plasmid pMM3015 transformed	This study

Table S3. The information of synthesized genes in the construction of engineered *E. coli* strains.

**Genes**	**Origins**	**Accession numbers**	**References**
ecTHIM	*Escherichia coli*	WP_001195634.1	[11]
mjIPK	*Methanocaldococcus jannaschii*	WP_010869535.1	[11]
idi	*Escherichia coli*	WP_001192820.1	[12]
ptmT4	*Streptomyces platensis*	AIW55562.1	[15]
cyS	*Streptomyces cattleya*	WP_014150548	[16]

Table S4. The data collection and refinement statistics of crystal structures.

	apo-CyS	CyS-GGPP-Mg^{2+}	CyS^{C59A}
Data collection			
Wavelength (Å)	0.97918	0.97918	1.54184
Space group	*F* 2 2 2	*F* 2 2 2	*F* 2 2 2
Unit cell			
a, b, c (Å)	100.08, 112.93, 138.22	100.67, 113.39, 138.31	100.28, 112.63, 137.38
α, β, γ (°)	90.000, 90.000, 90.000	90.000, 90.000, 90.000	90.000, 90.000, 90.000
Resolution range (Å)	65.85-2.00 (2.05-2.00) [b]	56.57-1.87 (1.91-1.87) [b]	29.30-2.30 (2.38-2.30) [b]
Unique reflections	34083 (4818)	32571 (3231)	98052 (17379)
R_{merge} [a]	0.148 (0.521)	0.080 (0.497)	0.096 (0.260)
$I/\sigma I$	13.6 (6.7)	19.7 (5.1)	18.3 (5.6)
Completeness (%)	99.6 (97.5)	100.0 (100.0)	99.5 (99.3)
Average redundancy	10.4 (8.2)	12.9 (12.8)	5.6 (4.6)
Structure refinement			
Resolution range (Å)	49.995-2.000	43.753-1.870	29.322-2.300
$R_{\text{work}}/R_{\text{free}}$	0.1749/0.2063	0.1812/0.2053	0.1877/0.2348
Number of protein atoms	2761	2774	2760
Number of water atoms	292	340	289
Bond lengths RMSD (Å)	0.0059	0.0064	0.0104
Bond angles RMSD (°)	0.78	0.90	0.99
Average B-factors ($Å^2$) for protein	17.14	24.54	19.18
Average B-factors ($Å^2$) for ligand		28.84	
Average B-factors ($Å^2$) for water	22.99	30.63	22.22
Most favored	99.14	98.01	97.43
Additional allowed	0.86	1.99	2.57
Outliers	0	0	0
Protein Data Bank entry	7Y50	7Y88	7Y87

[a] $R_{merge} = \sum_{hkl}\sum_i |I_i(hkl) - \langle I(hkl)\rangle| / \sum_{hkl}\sum_i I_i(hkl)$, where $I_i(hkl)$ is the *i*th observation of reflection *hkl*, and $\langle I(hkl)\rangle$ is the weighted average intensity for all observations of that reflection *hkl*.
[b] Numbers in parentheses are values for the highest-resolution bin.

Table S5. The ^{1}H NMR (600 MHz for **1** and 700 MHz for **2-6**) data (δ_H, J in Hz) of **1-6** in $CDCl_3$.

position	**1**	**2**	**3**	**4**	**5**	**6**
1	1.92, s	1.57, br s*	1.61, m;<br>1.31, m	2.21, d (16.0);<br>1.76, m	5.39, s	1.83, d (13.2);<br>1.25, m*
2	-	2.00, br q, (9.8)	2.31, m	-	-	
3	-	-	-	-	2.49, m (7.6)	1.64, d (10.0)
4	1.77, m;<br>1.38, m	1.72, m;<br>1.53, m	2.50, dd (12.9, 3.8);<br>1.58, m	2.34, m;<br>2.07, m	1.82, m;<br>1.18, m	-
5	2.26, m;<br>2.09, m	2.27, m;<br>2.09, m	2.36, m;<br>1.99, m	2.13, m;<br>1.77, m	1.70, m;<br>1.18, m	5.34, m
6	1.39, m		5.31, dd (11.8, 2.9)	-	-	2.46, d (15.3);<br>1.67, d (15.3)
7	-		-	1.91, m	1.75, m	1.10, m
8	1.66, m;<br>1.13, m	2.20, m;<br>2.15, m	1.94, m	1.64, m;<br>1.46, m	1.98, m;<br>1.39, m*	1.25, m*;<br>1.15, m
9	1.86, m;<br>1.28, m	1.51, m;<br>1.39, m	1.52, m;<br>1.14, m	2.09, m;<br>1.45, m	1.85, m;<br>1.45, m	1.53, m;<br>1.26, m
10	2.05, m	1.79, m	2.09, m	2.01, dm (11.9)	1.57, m	1.73, m
11	-	-	-	-	-	-
12	1.55, m*;<br>1.49, m	1.50, m	1.53, m;<br>1.49, m	1.48, m;<br>1.26, m	1.47, m;<br>1.39, m*	1.44, m;<br>1.35, m
13	1.55, m*;<br>1.33, m	1.41, m	1.44, m*;<br>1.39, m	1.36, m;<br>1.24, m	1.58, m;<br>1.34, m	1.51, m;<br>1.24, m

14	1.01, br s	1.20, d (3.2)	1.44, m*	1.29, m	1.25, m	1.50, m
15	-	-	-	-	-	-
16	0.99, s	0.93, s	1.02, s*	0.71, s	0.86, s	0.95, s
17	0.97, s	1.01, s	1.02, s*	0.98, s	1.12, s	0.97, s
18	1.04, s	1.22, s	1.16, s	1.12, s	1.10, s	1.27, s
19	0.89, d (6.5)	1.57, s*	1.51, s	0.84, d (8.3)	0.83, d (7.4)	1.01, d (6.1)
20	0.74, s	0.92, s	4.82, s;<br>4.76, s	1.65, s	1.08, d (7.1)	1.82, s

*These signals overlapped with each other.
All signals were assigned based on 2D NMR experiments.

Table S6. The ^{13}C NMR (150 MHz for **1** and 175 MHz for **2-6**) data (δ_C, type) of **1-6** in $CDCl_3$.

position	**1**	**2**	**3**	**4**	**5**	**6**
1	44.7, CH_2	43.7, CH_2	44.8, CH_2	37.3, CH_2	130.3, CH	58.8, CH_2
2	132.9, C	51.2, CH	53.9, CH	137.1, C	144.5, C	55.7, C
3	137.7, C	48.1, C	149.4, C	129.8, C	37.6, CH	58.1, CH
4	25.3, CH_2	43.3, CH_2	38.9, CH_2	37.0, CH_2	31.3, CH_2	146.3, C
5	30.3, CH_2	35.7, CH_2	28.7, CH_2	41.0, CH_2	44.3, CH_2	124.5, CH
6	45.9, CH	127.4, C	123.4, CH	58.5, C	56.6, C	43.7, CH_2
7	42.3, C	140.5, C	136.5, C	50.9, CH	41.8, CH	37.0, CH
8	36.6, CH_2	22.2, CH_2	41.3, CH_2	35.1, CH_2	33.0, CH_2	28.8, CH_2
9	27.8, CH_2	27.3, CH_2	33.1, CH_2	38.6, CH_2	32.5, CH_2	25.4, CH_2
10	44.1, CH	42.1, CH	45.5, CH	46.9, CH	47.4, CH	44.5, CH
11	50.3, C	50.8, C	48.2, C	42.7, C	45.2, C	48.7, C
12	39.0, CH_2	40.2, CH_2	41.0, CH_2	40.8, CH_2	40.0, CH_2	42.2, CH_2
13	40.2, CH_2	41.9, CH_2	42.2, CH_2	40.0, CH_2	41.2, CH_2	39.4, CH_2
14	69.0, CH	67.8, CH	72.9, CH	64.1, CH	57.8, CH	63.3, CH
15	41.3, C	42.9, C	43.0, C	44.4, C	42.5, C	42.3, C
16	25.1, CH_3	26.0, CH_3	24.7, CH_3	27.7, CH_3	26.1, CH_3	24.8, CH_3
17	30.0, CH_3	31.7, CH_3	33.0, CH_3	31.1, CH_3	33.5, CH_3	29.8, CH_3
18	31.5, CH_3	31.8, CH_3	31.6, CH_3	34.5, CH_3	29.9, CH_3	33.4, CH_3
19	14.1, CH_3	14.0, CH_3	19.0, CH_3	15.5, CH_3	22.1, CH_3	22.9, CH_3
20	18.5, CH_3	24.1, CH_3	112.1, CH_2	13.8, CH_3	21.8, CH_3	19.5, CH_3

Table S7. Top 10 homologues of CyS from the DALI alignment.

	PDB ID	Description	Z	R.m.s.d (Å)	Identity (%)	Solved Method
1	4okm-A	Selinadiene synthase	43.2	1.9	32	X-RAY
2	6tbd-A	Terpene synthase	40.9	1.7	41	X-RAY
3	7ofl-A	Terpene synthase	36.8	2.0	22	X-RAY
4	5a0j-A	Labdane-related diterpene synthase	33.8	2.1	23	X-RAY
5	5dz2-A	Germacradienlo/Geosmin synthase	33.6	2.2	21	X-RAY
6	3kb9-A	Epi-isozizaene synthase	33.4	2.7	21	X-RAY
7	6q4s-A	Pentalenene synthase	32.8	2.1	22	X-RAY
8	2oa6-D	Aristolochene synthase	32.6	2.6	18	X-RAY
9	4la6-A	2-methylisoborneol synthase	32.6	2.3	20	X-RAY
10	5nx7-A	Pentalenene synthase	32.5	2.3	23	X-RAY

Table S8. Isotopic labeling experiments with CyS^{C59A}.

No.	substrates	enzymes	results shown in
1	FPP + (1-^{13}C)IPP[17]	GGPPS + CyS^{C59A}	Figure S34B
2	FPP + (2-^{13}C)IPP[18]	GGPPS + CyS^{C59A}	Figure S34C
3	FPP + (3-^{13}C)IPP[17]	GGPPS + CyS^{C59A}	Figure S34K
4	FPP + (4-^{13}C)IPP[17]	GGPPS + CyS^{C59A}	Figure S34L
5	(1-^{13}C)FPP[19] + IPP	GGPPS + CyS^{C59A}	Figure S34D
6	(2-^{13}C)FPP[19] + IPP	GGPPS + CyS^{C59A}	Figure S34E
7	(3-^{13}C)FPP[19] + IPP	GGPPS + CyS^{C59A}	Figure S34F
8	(4-^{13}C)FPP[19] + IPP	GGPPS + CyS^{C59A}	Figure S34G
9	(5-^{13}C)FPP[19] + IPP	GGPPS + CyS^{C59A}	Figure S34H
10	(6-^{13}C)FPP[19] + IPP	GGPPS + CyS^{C59A}	Figure S34I
11	(7-^{13}C)FPP[19] + IPP	GGPPS + CyS^{C59A}	Figure S35M
12	(8-^{13}C)FPP[19] + IPP	GGPPS + CyS^{C59A}	Figure S35N
13	(9-^{13}C)FPP[19] + IPP	GGPPS + CyS^{C59A}	Figure S35O
14	(10-^{13}C)FPP[19] + IPP	GGPPS + CyS^{C59A}	Figure S35P
15	(11-^{13}C)FPP[19] + IPP	GGPPS + CyS^{C59A}	Figure S35Q
16	(12-^{13}C)FPP[19] + IPP	GGPPS + CyS^{C59A}	Figure S35R
17	(9-^{13}C)GPP[20] + IPP	GGPPS + CyS^{C59A}	Figure S35S
18	(14-^{13}C)FPP[19] + IPP	GGPPS + CyS^{C59A}	Figure S35T
19	(15-^{13}C)FPP[19] + IPP	GGPPS + CyS^{C59A}	Figure S35U
20	FPP + (5-^{13}C)IPP[21]	GGPPS + CyS^{C59A}	Figure S35V
21	(2-^{2}H, 3-^{13}C)FPP[22] + IPP	GGPPS + CyS^{C59A}	Figure S36C
22	(2-^{2}H, 3-^{13}C)GGPP[17]	CyS^{C59A}	Figure S36F
23	DMAPP + (E)-(4-^{2}H, 4-^{13}C)IPP[14]	GGPPS + CyS^{C59A}	Figure S37C
24	DMAPP + (Z)-(4-^{2}H, 4-^{13}C)IPP[14]	GGPPS + CyS^{C59A}	Figure S37D

Figure S1. The SDS-PAGE and Mg^{2+}-binding network. (A) The SDS-PAGE of CyS and CyS^{C59A}. The calculated molecule weight of CyS or CyS^{C59A} is ~42.0 kDa. (B) The Mg^{2+}-binding network in CyS-GGPP-$^{Mg2+}$ structure. Residues are shown as yellow sticks, and GGPP is shown as magenta sticks. The Mg^{2+} ions are shown as green spheres.

A

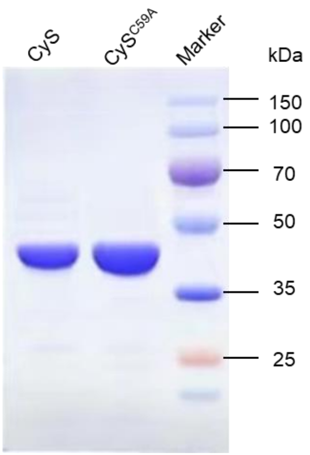

B

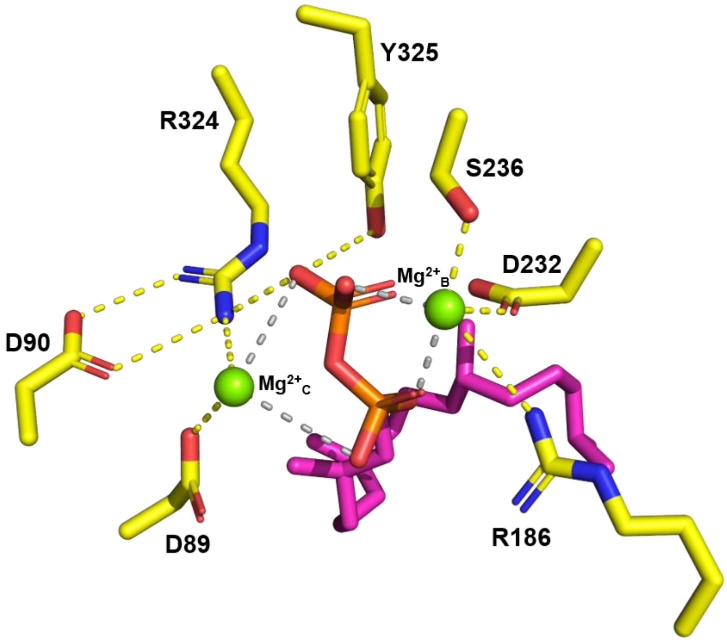

Figure S2. The construction of engineered *E. coli* strains.

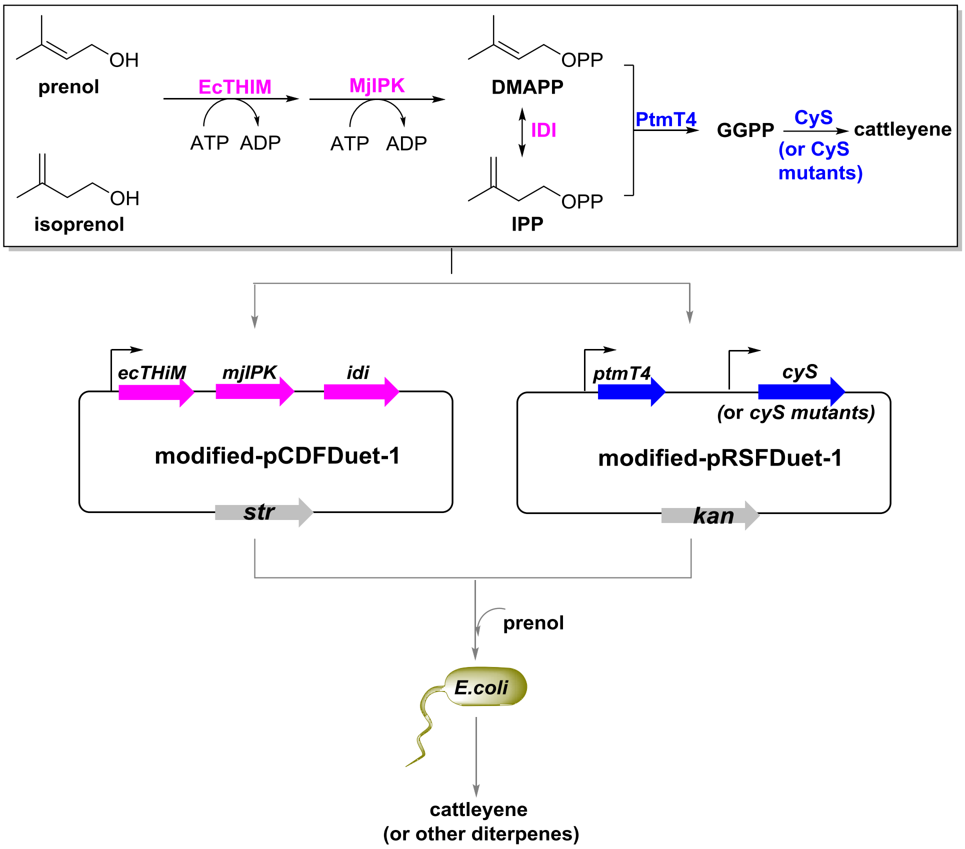

Figure S3. GC-MS analysis (extracted ion chromatogram at *m/z* 272) of the products of other CyS mutants.

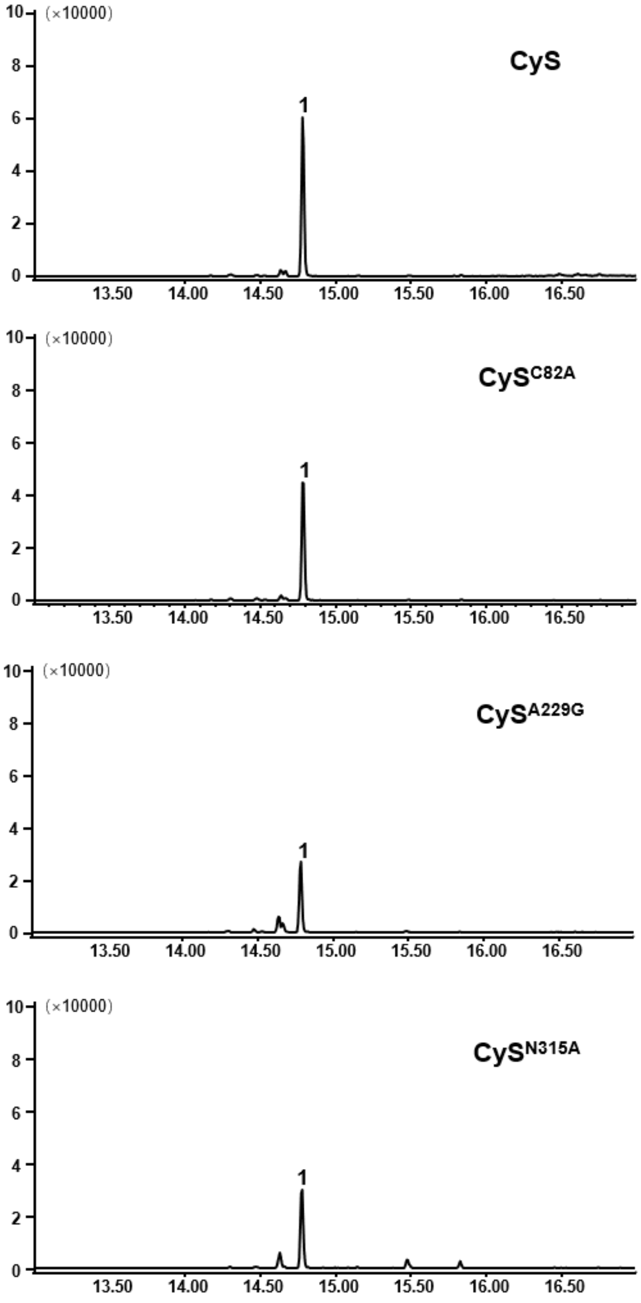

Figure S4. Mass spectra of compounds **1**-**6** from GC-MS analysis.

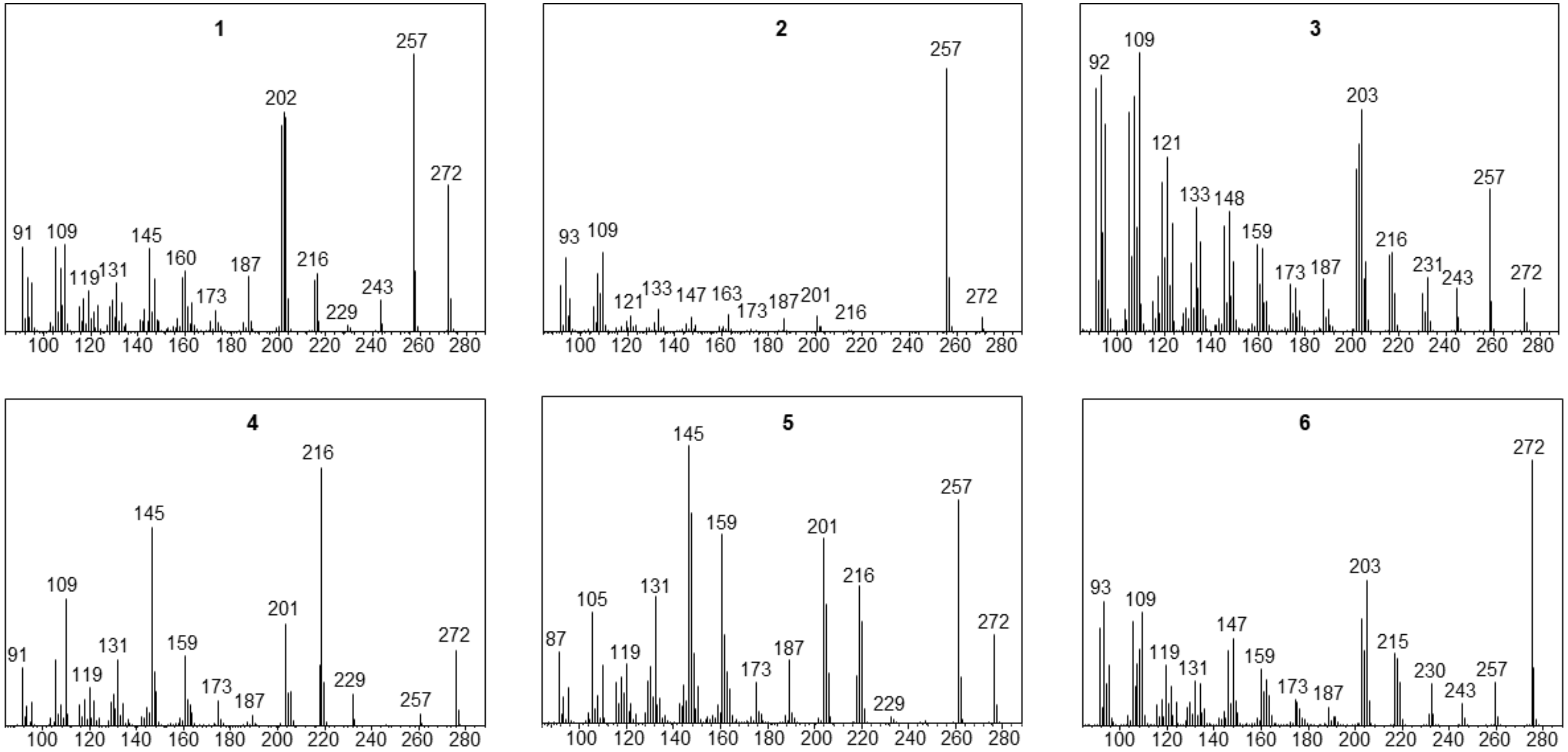

Figure S5. The key 2D NMR correlations of new diterpene variants.

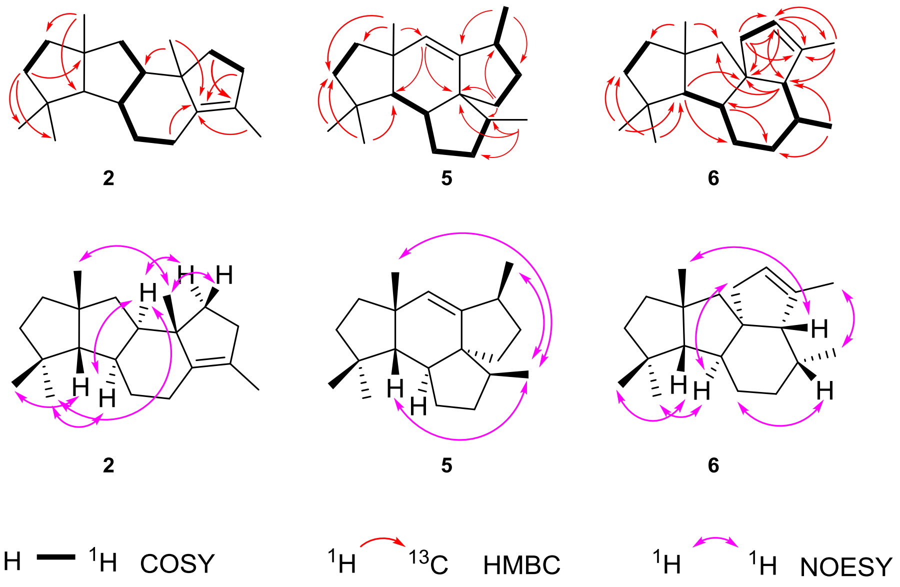

Figure S6. The proposed biosynthetic pathways of **1**-**6** (shown in black squares).

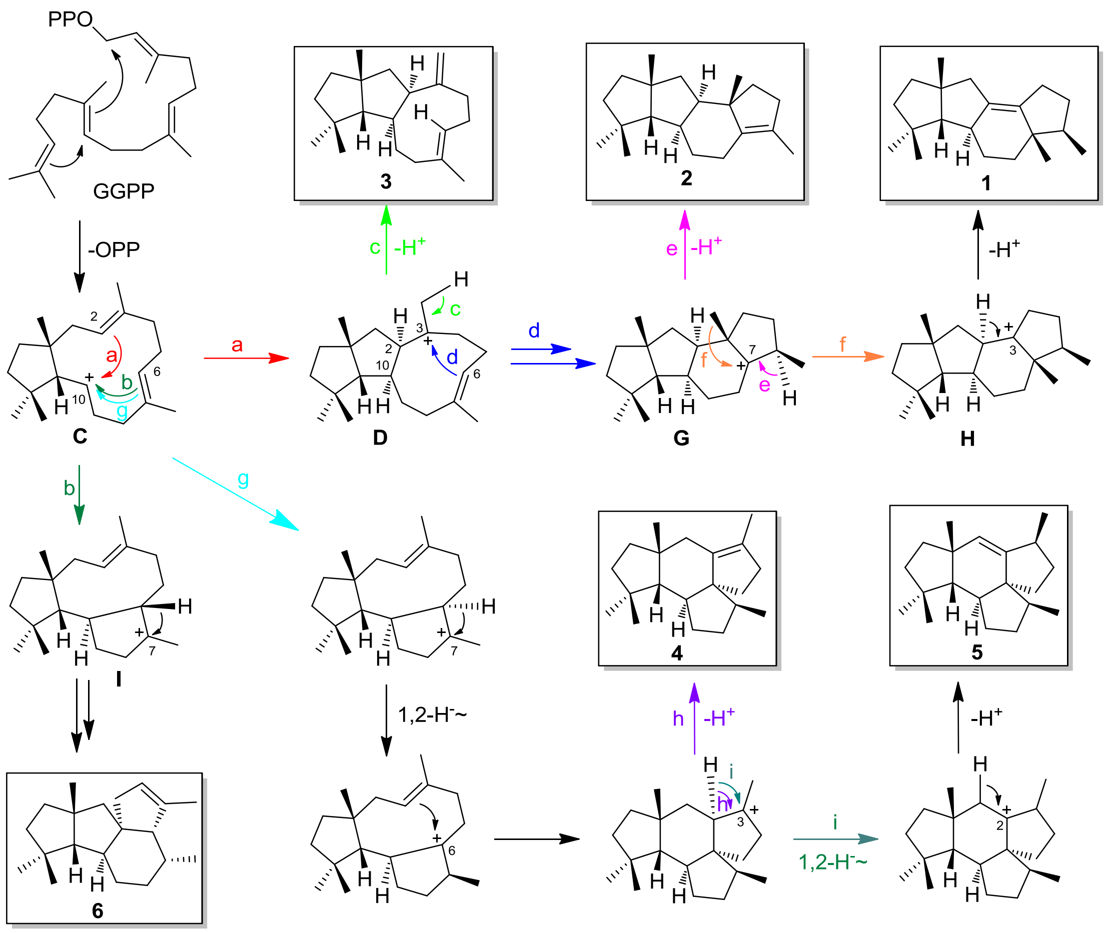

Figure S7. The ^{1}H NMR (600 MHz) and ^{13}C NMR (125 MHz) spectrum of compound **1** in $CDCl_3$.

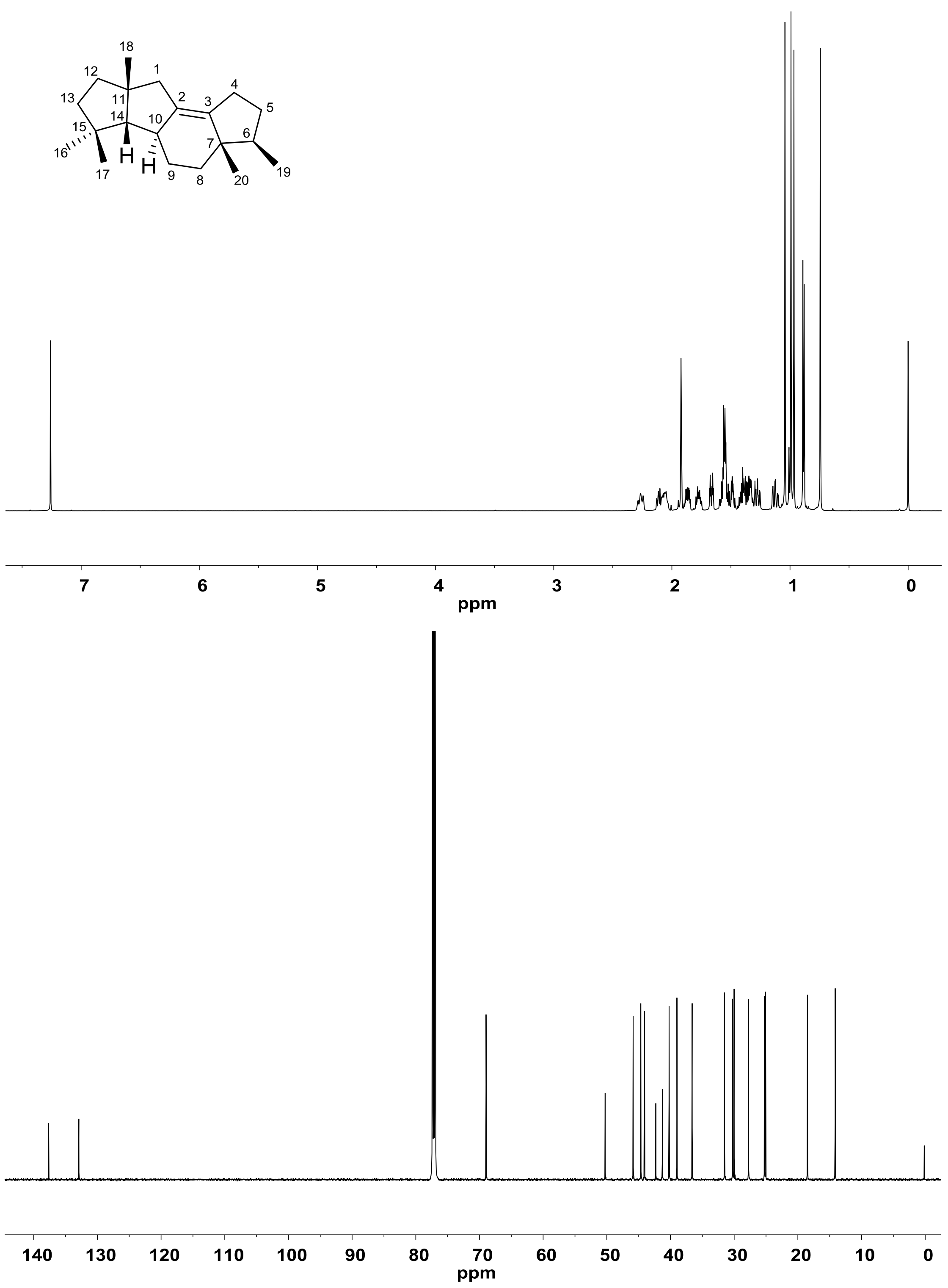

Figure S8. The ^{1}H NMR (700 MHz) and ^{13}C NMR (175 MHz) spectrum of compound **2** in $CDCl_3$.

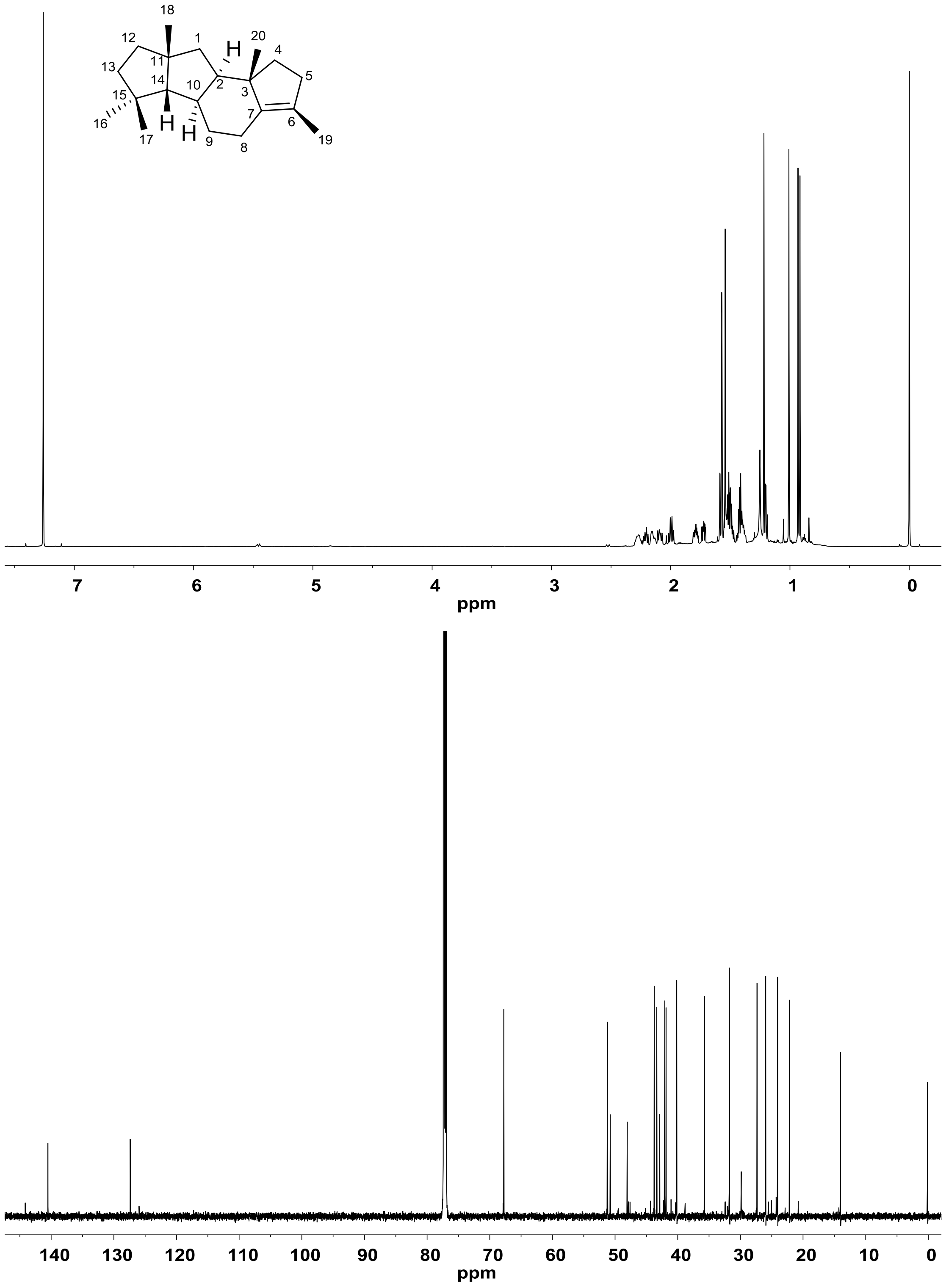

Figure S9. The COSY spectrum of compound **2** in $CDCl_3$.

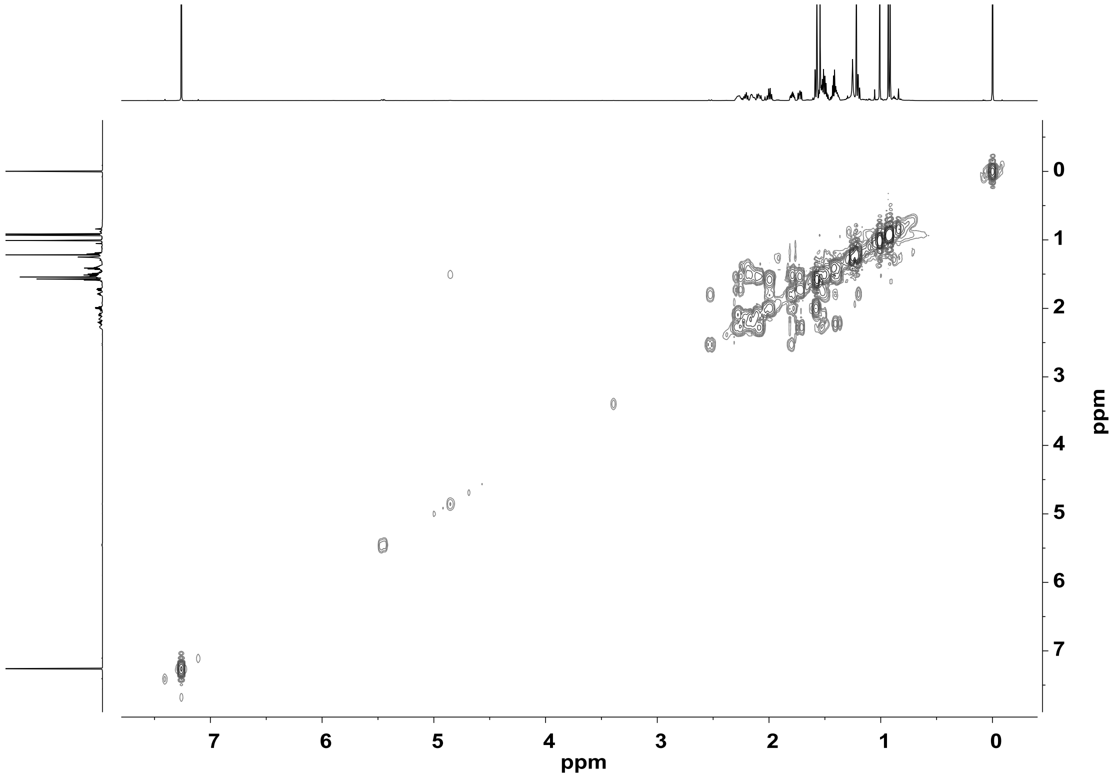

Figure S10. The HSQC spectrum of compound **2** in $CDCl_3$.

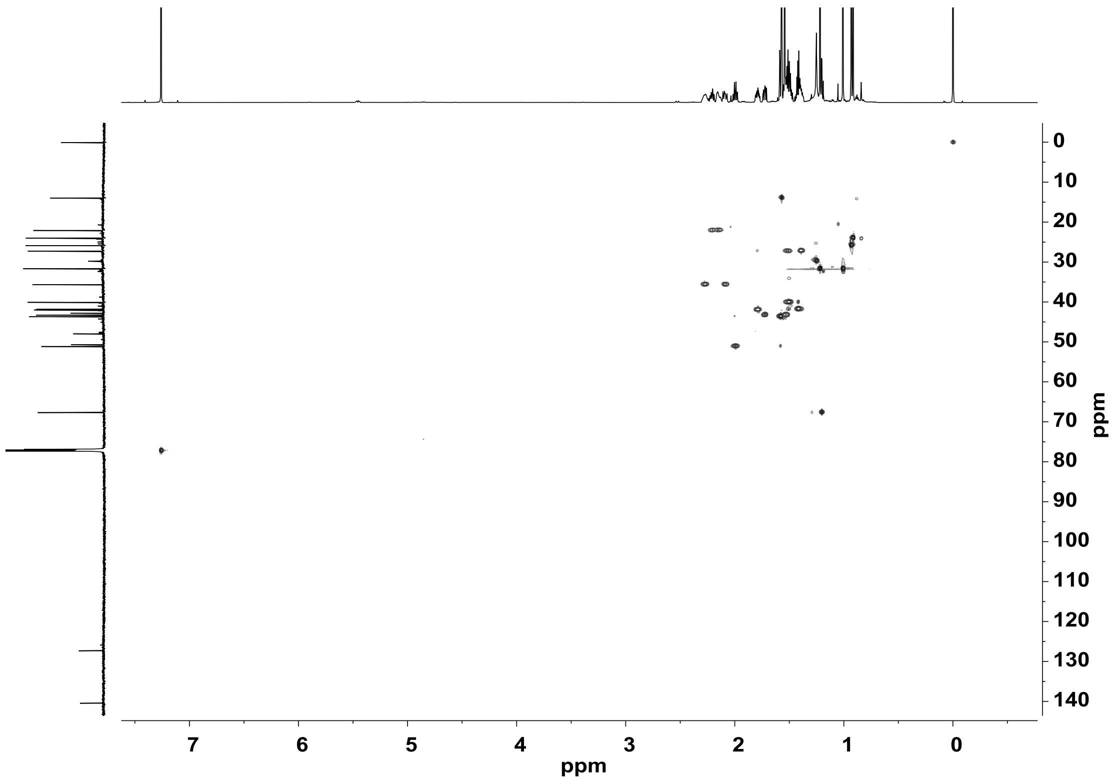

Figure S11. The HMBC spectrum of compound **2** in $CDCl_3$.

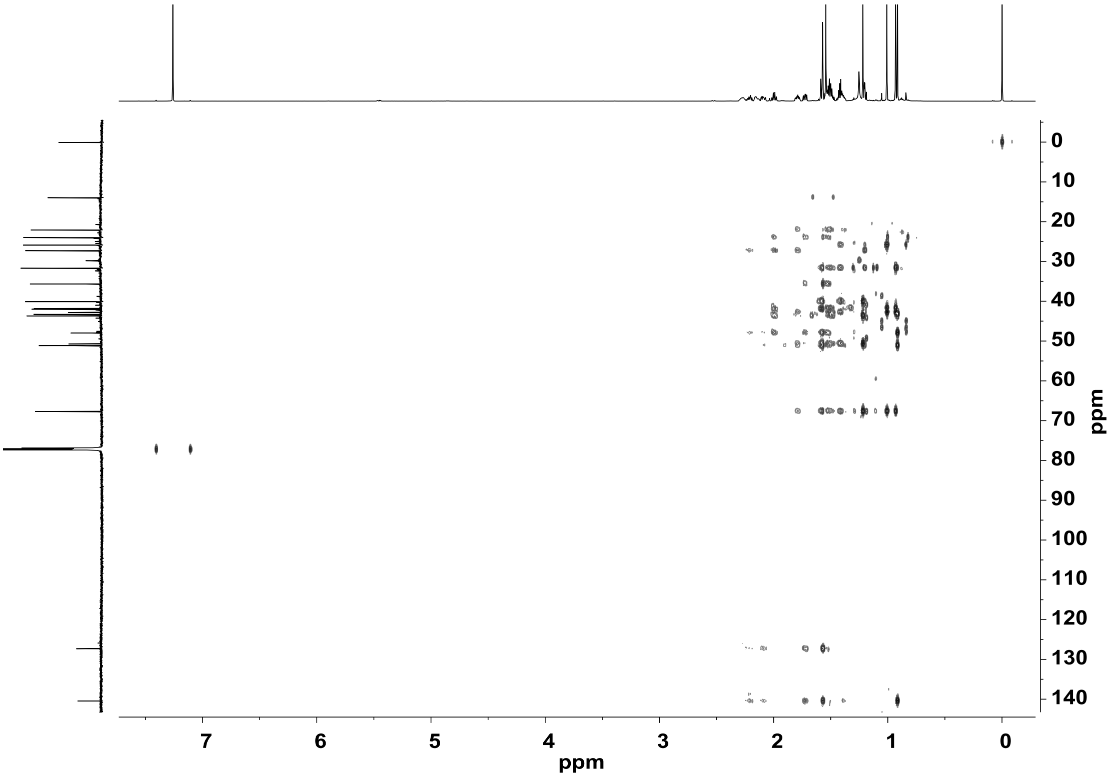

Figure S12. The DEPT 135° spectrum of compound **2** in $CDCl_3$.

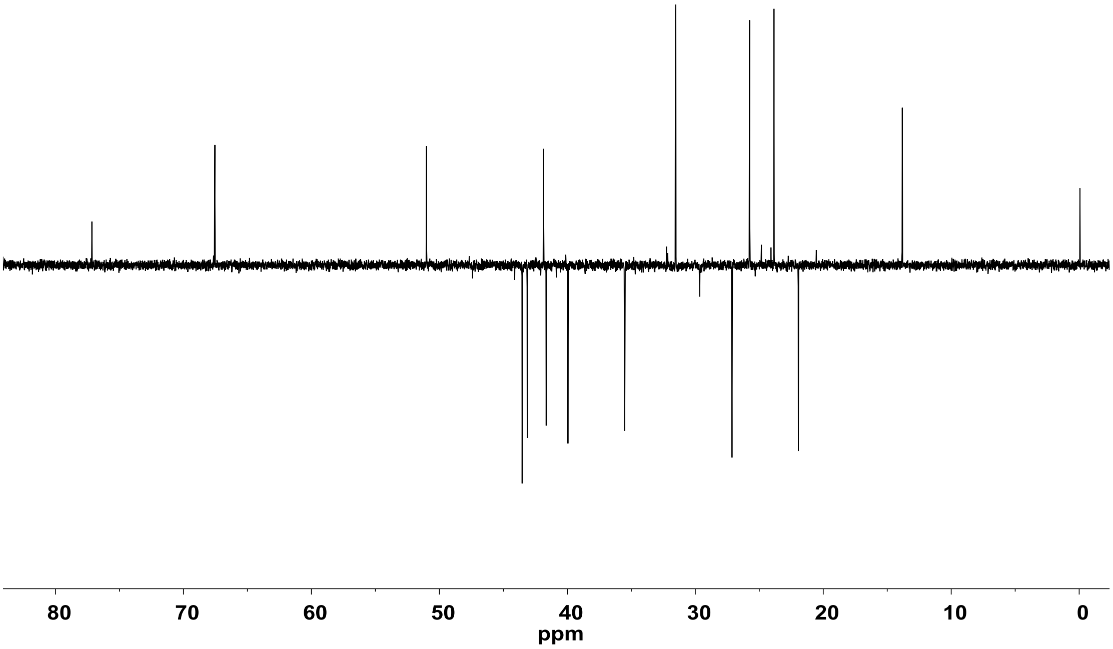

Figure S13. The NOESY spectrum of compound **2** in $CDCl_3$.

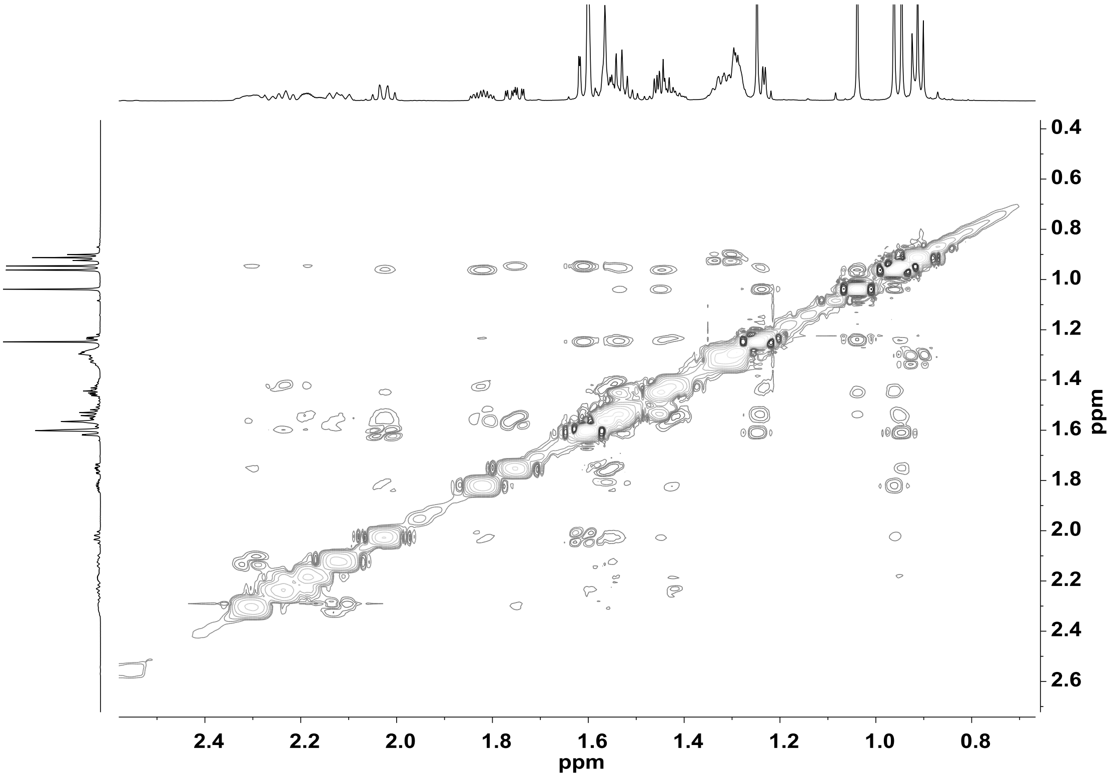

Figure S14. The IR spectrum of compound **2**.

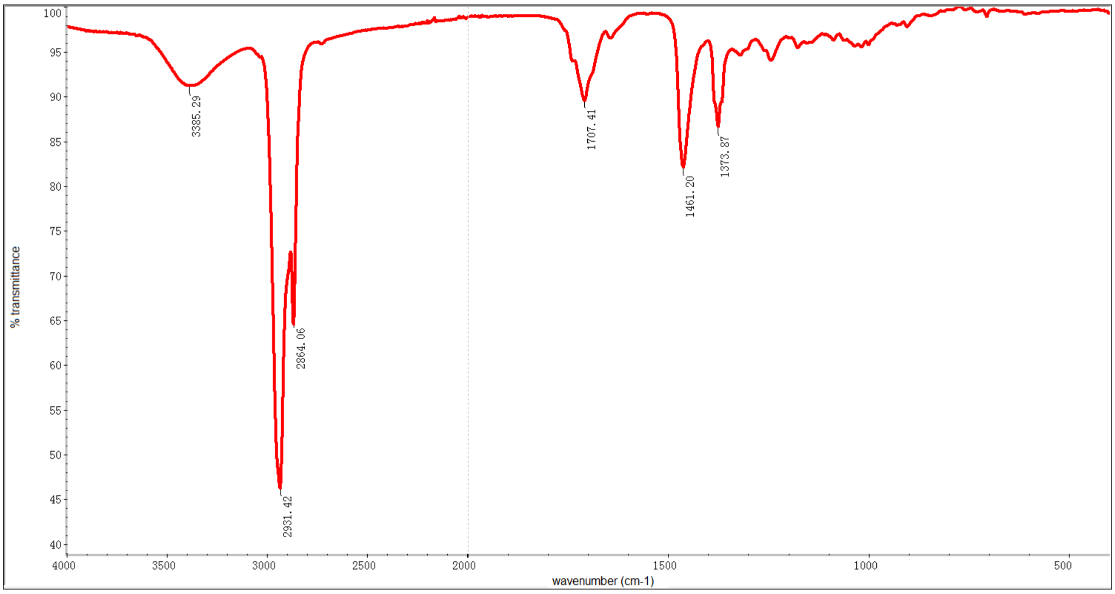

Figure S15. The HRMS (EI) spectrum of compound **2**.

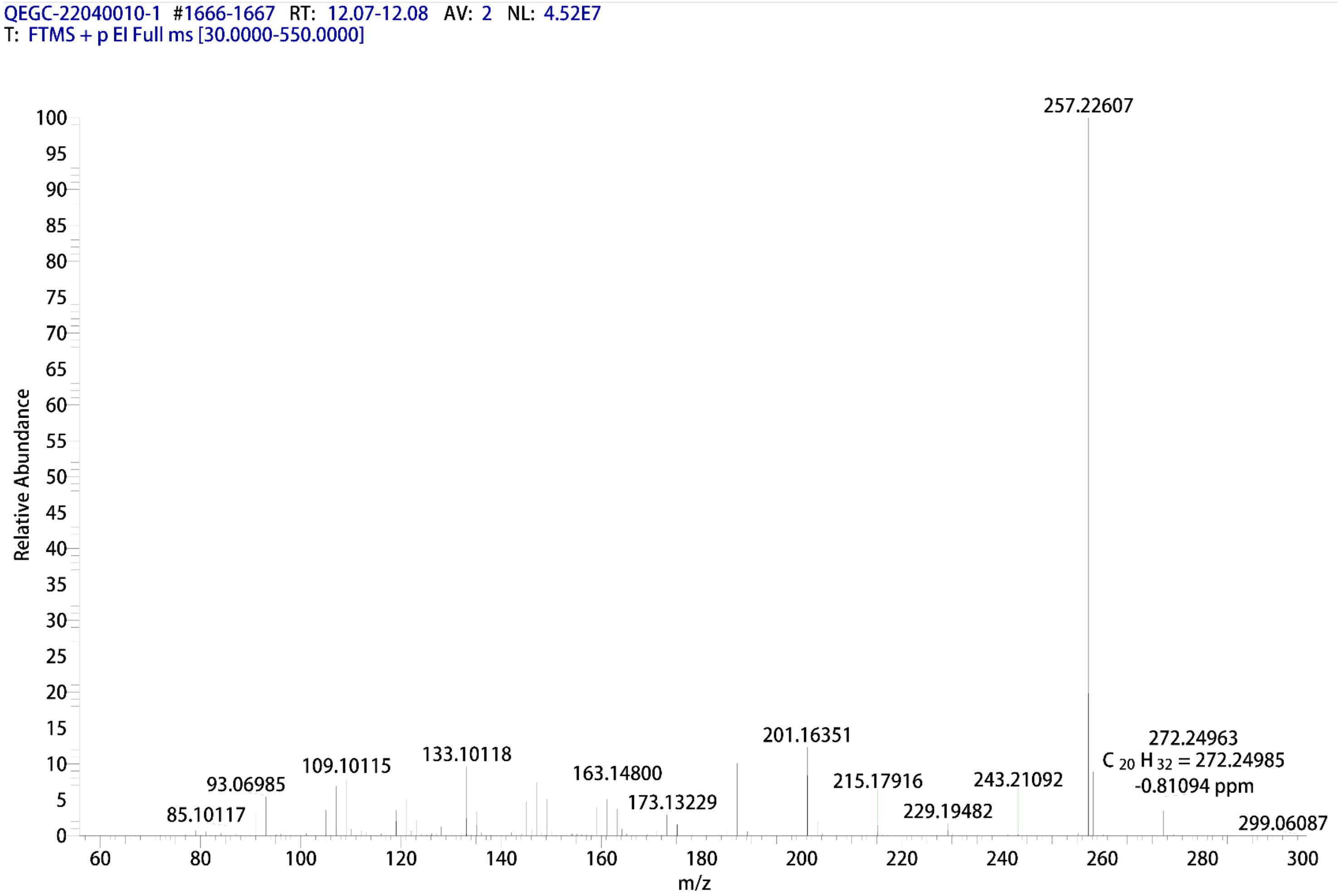

Figure S16. The ^{1}H NMR (700 MHz) and ^{13}C NMR (175 MHz) spectrum of compound **3** in $CDCl_3$.

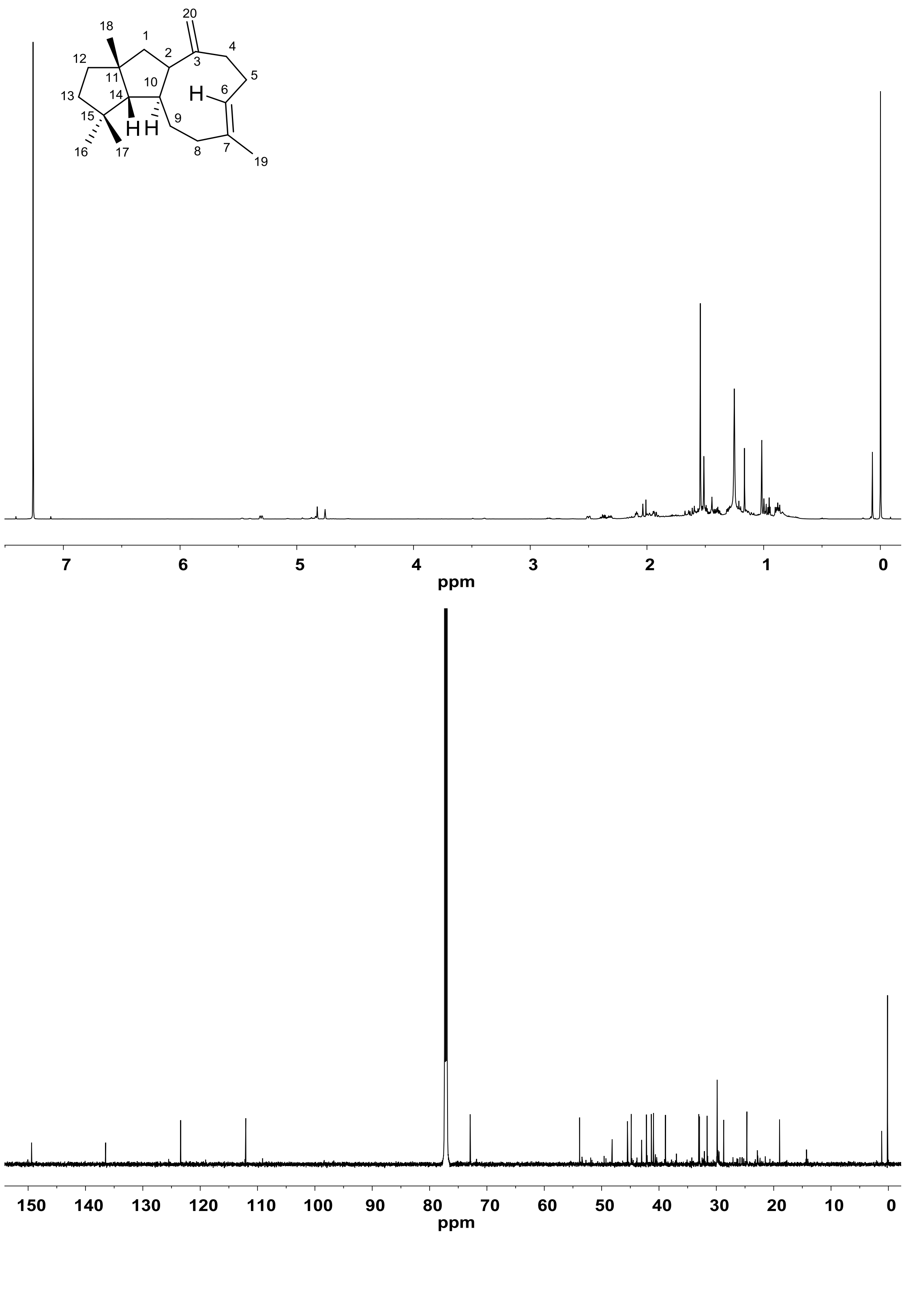

Figure S17. The ^{1}H NMR (700 MHz) and ^{13}C NMR (175 MHz) spectrum of compound **4** in $CDCl_3$

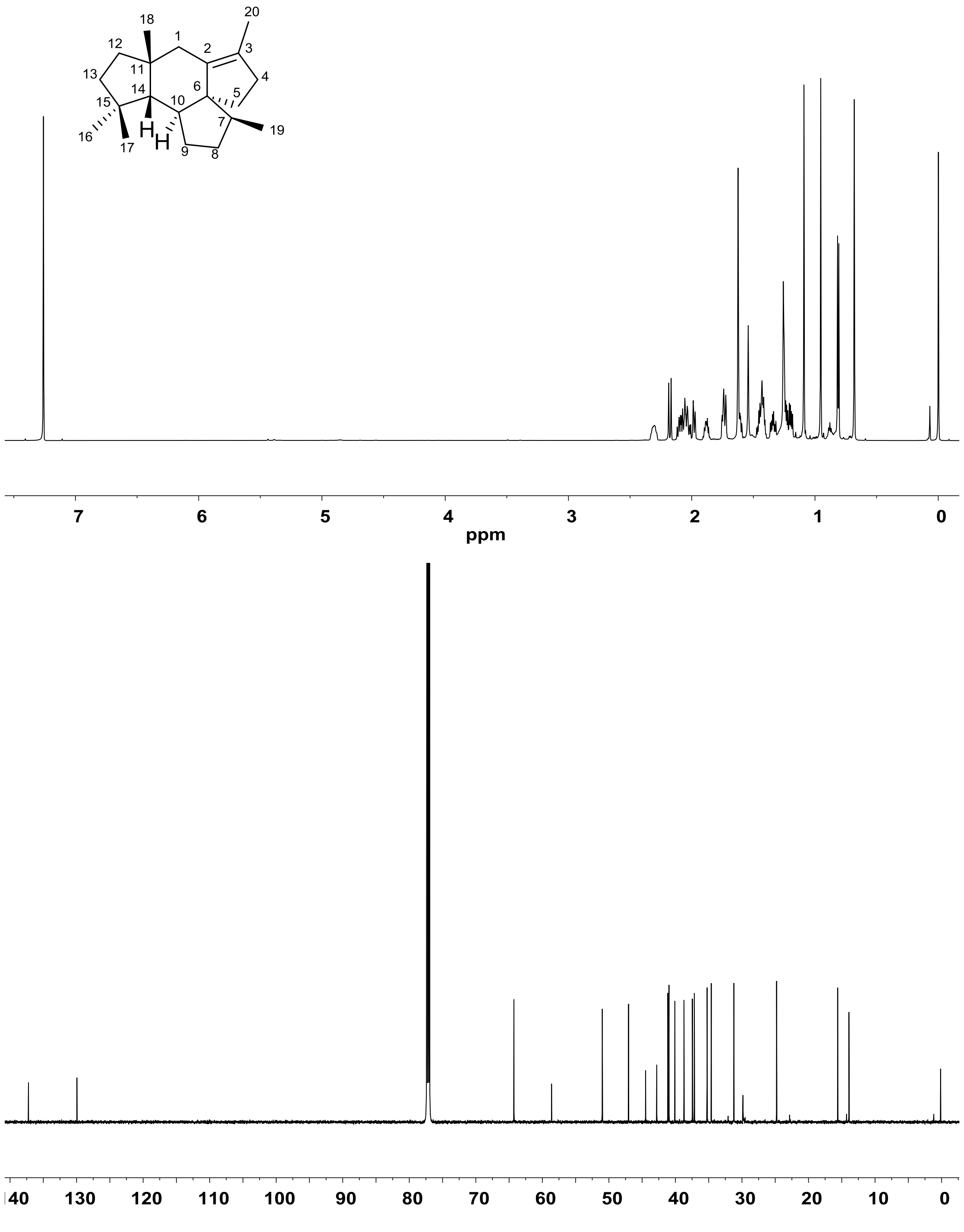

Figure S18. The ^{1}H NMR (700 MHz) and ^{13}C NMR (175 MHz) spectrum of compound **5** in $CDCl_3$

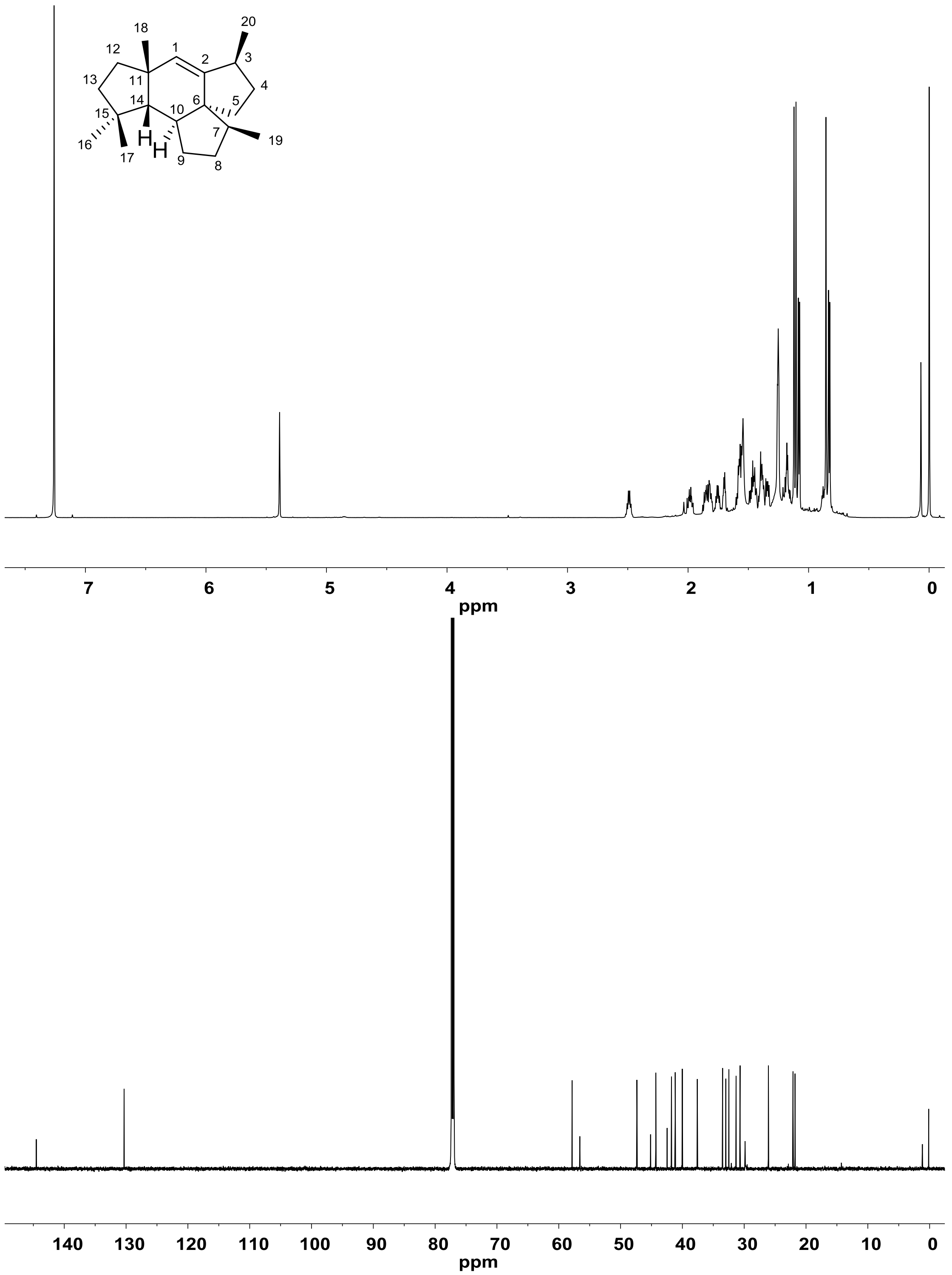

Figure S19. The COSY spectrum of compound **5** in $CDCl_3$.

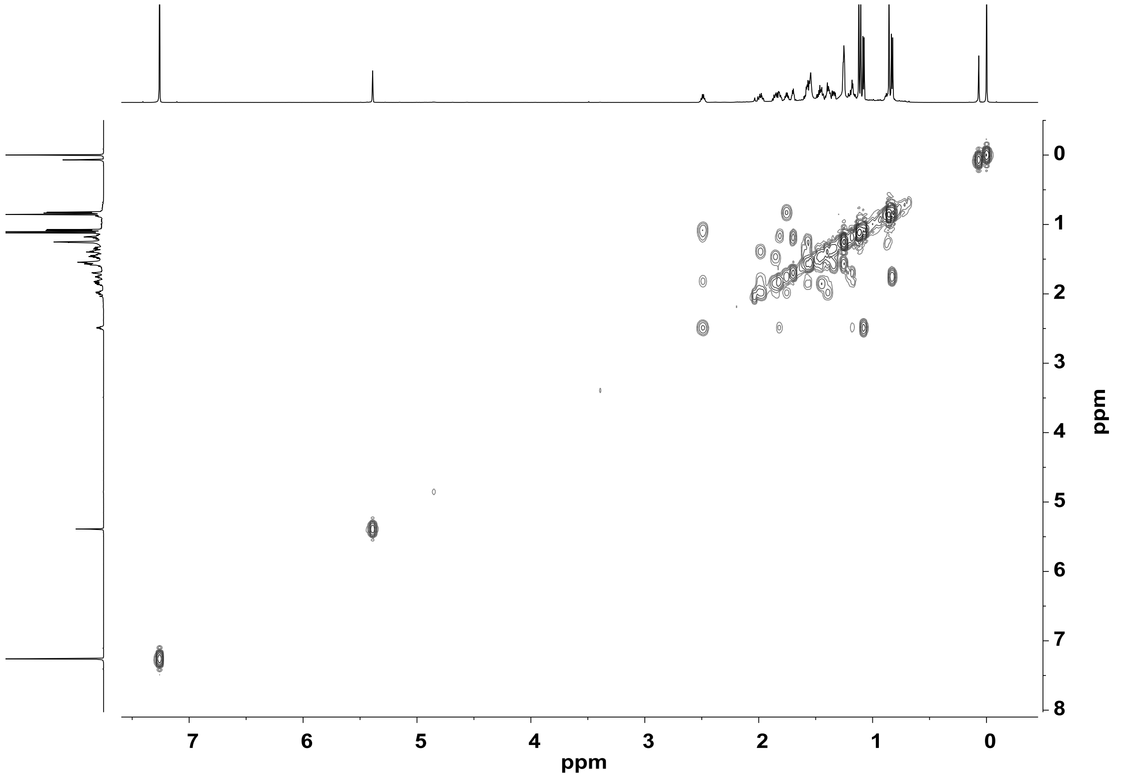

Figure S20. The HSQC spectrum of compound **5** in $CDCl_3$.

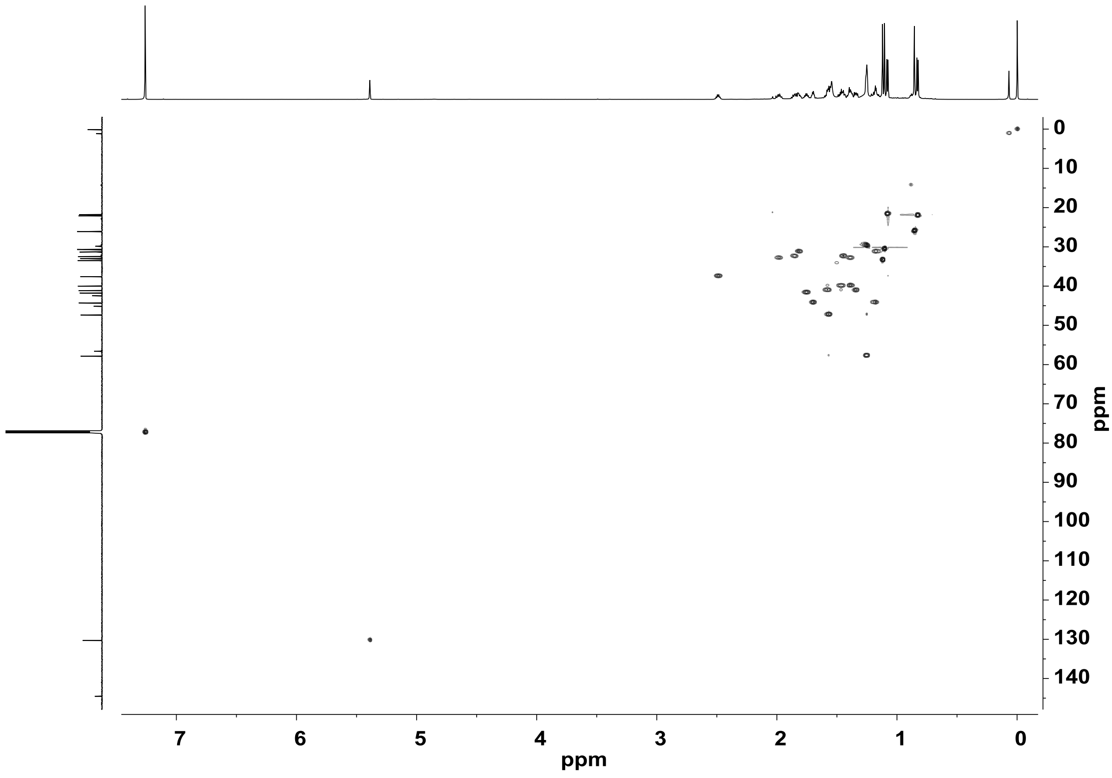

Figure S21. The HMBC spectrum of compound **5** in $CDCl_3$.

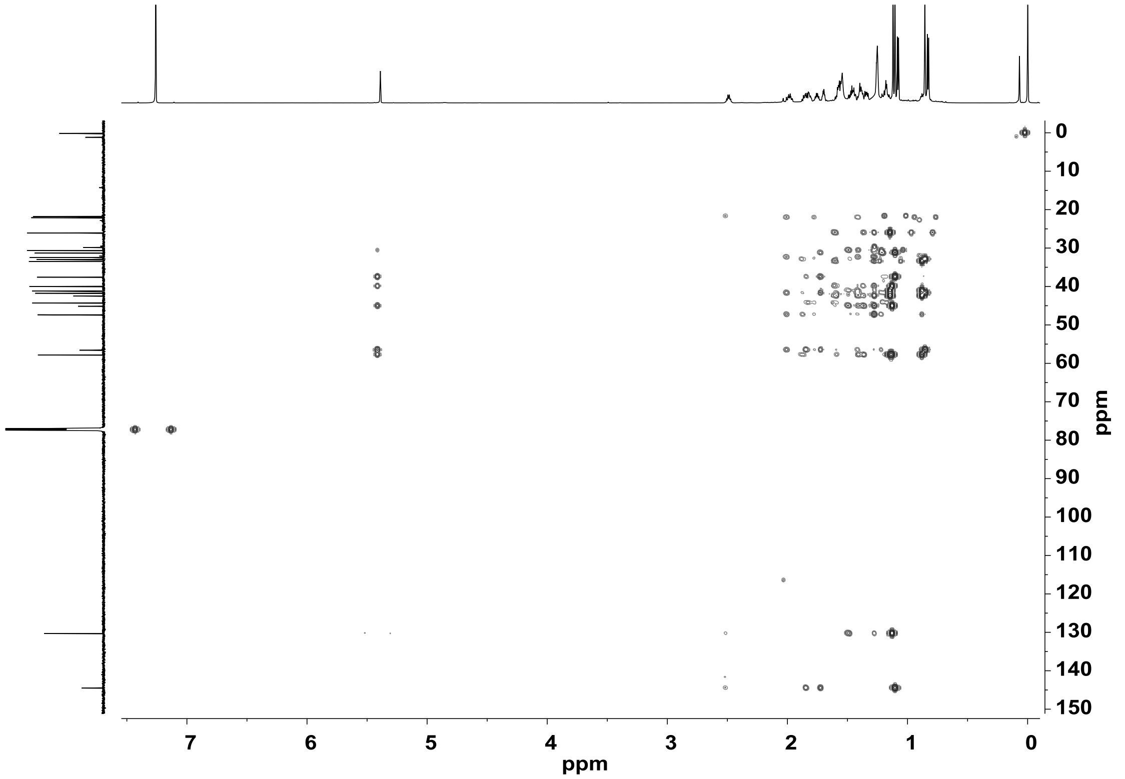

Figure S22. The DEPT135° spectrum of compound **5** in $CDCl_3$.

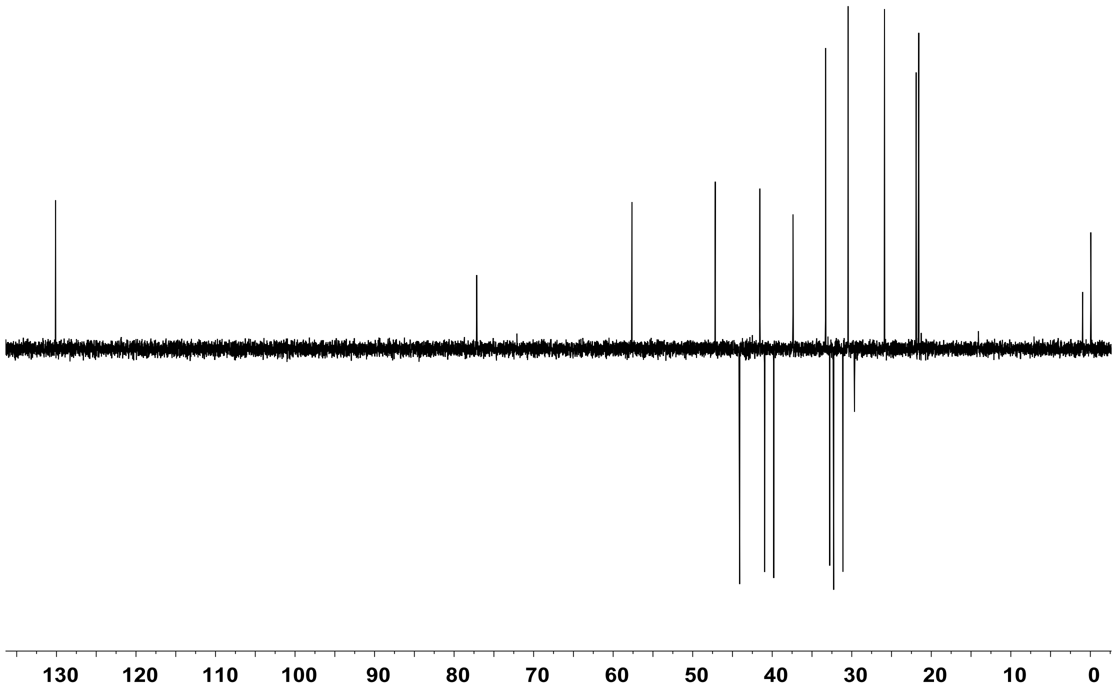

Figure S23. The 1D NOE spectrum of compound **5** in $CDCl_3$ by saturating H-19 resonance.

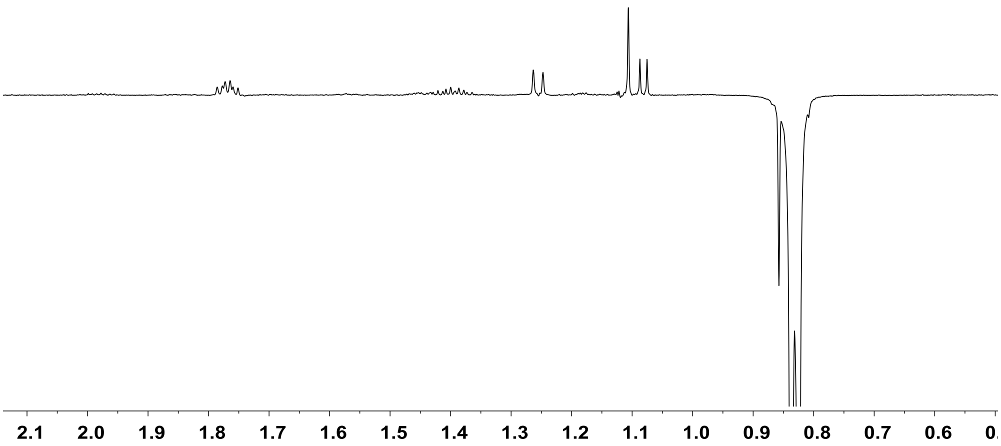

Figure S24. The IR spectrum of compound **5**.

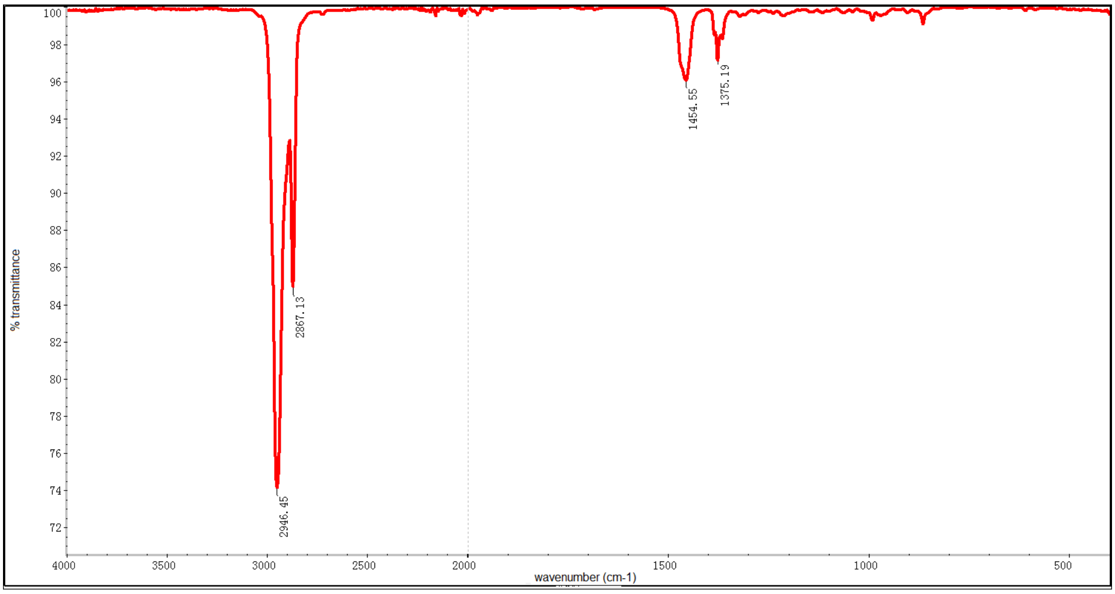

Figure S25. The HRMS (EI) spectrum of compound **5**.

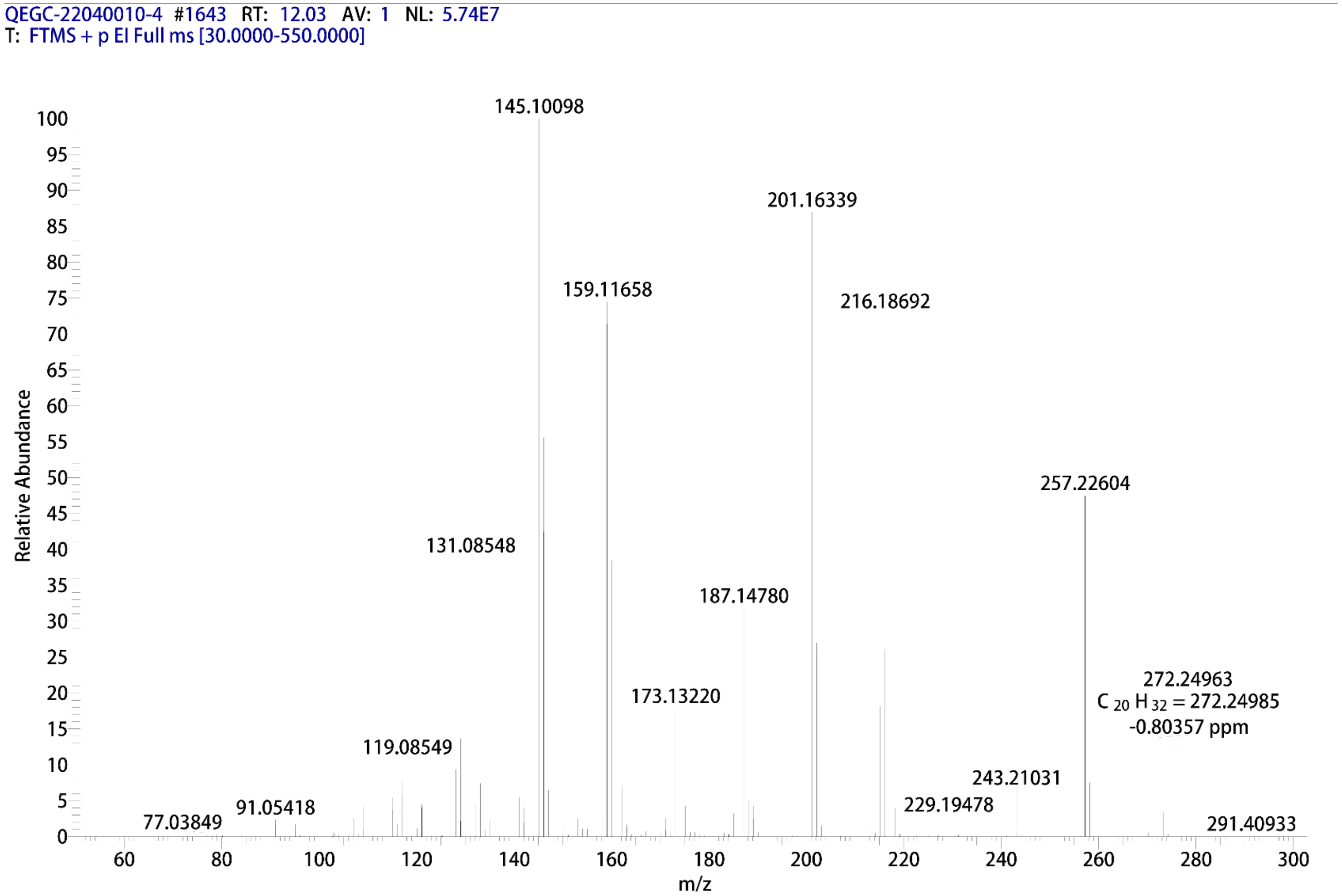

Figure S26. The ^{1}H NMR (700 MHz) and ^{13}C NMR (175 MHz) spectrum of compound **6** in $CDCl_3$.

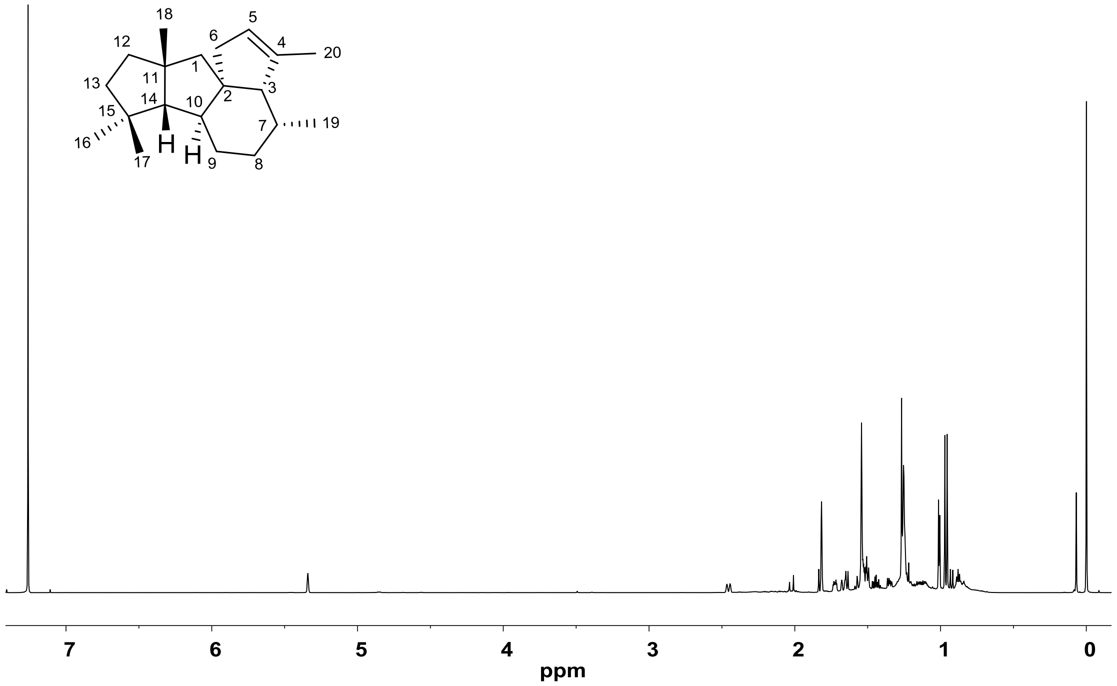

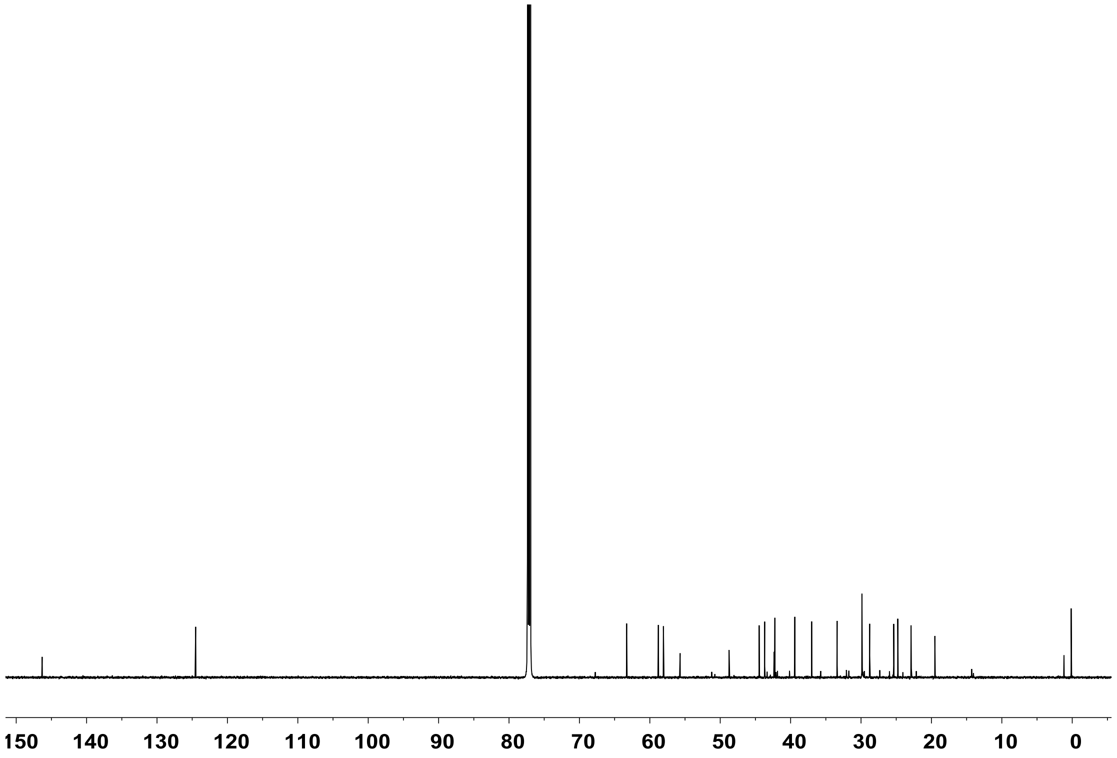

Figure S27. The COSY spectrum of compound **6** in $CDCl_3$.

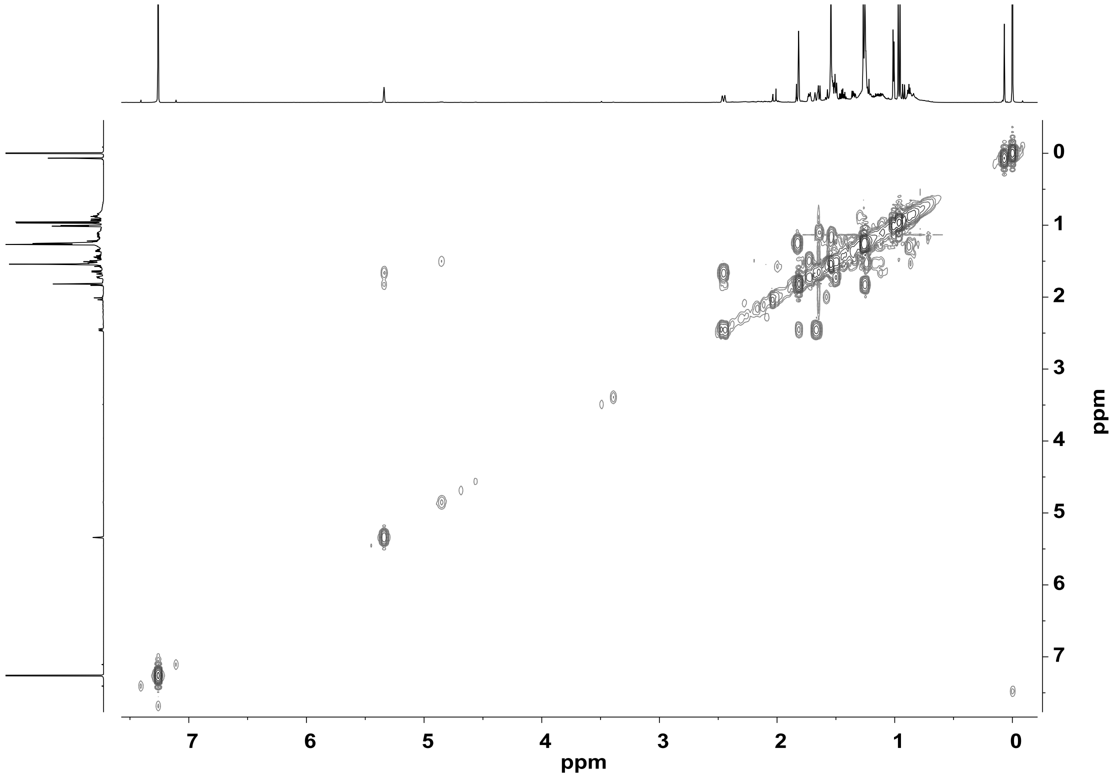

Figure S28. The HSQC spectrum of compound **6** in $CDCl_3$.

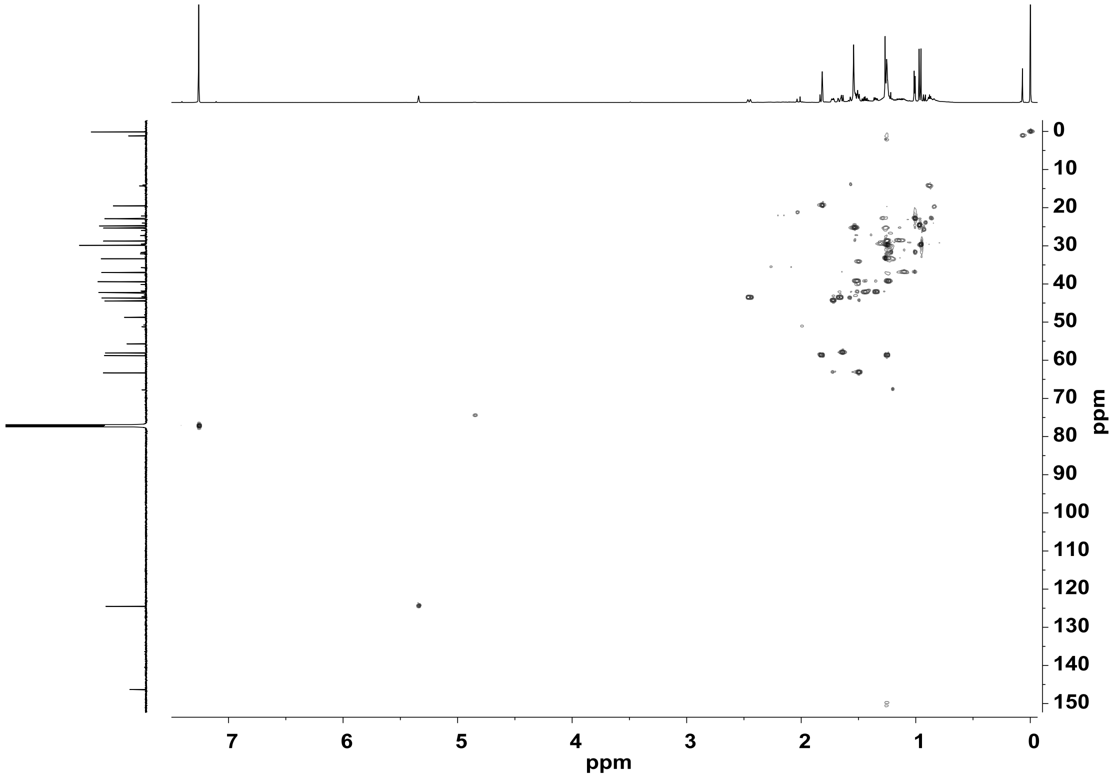

Figure S29. The HMBC spectrum of compound **6** in $CDCl_3$.

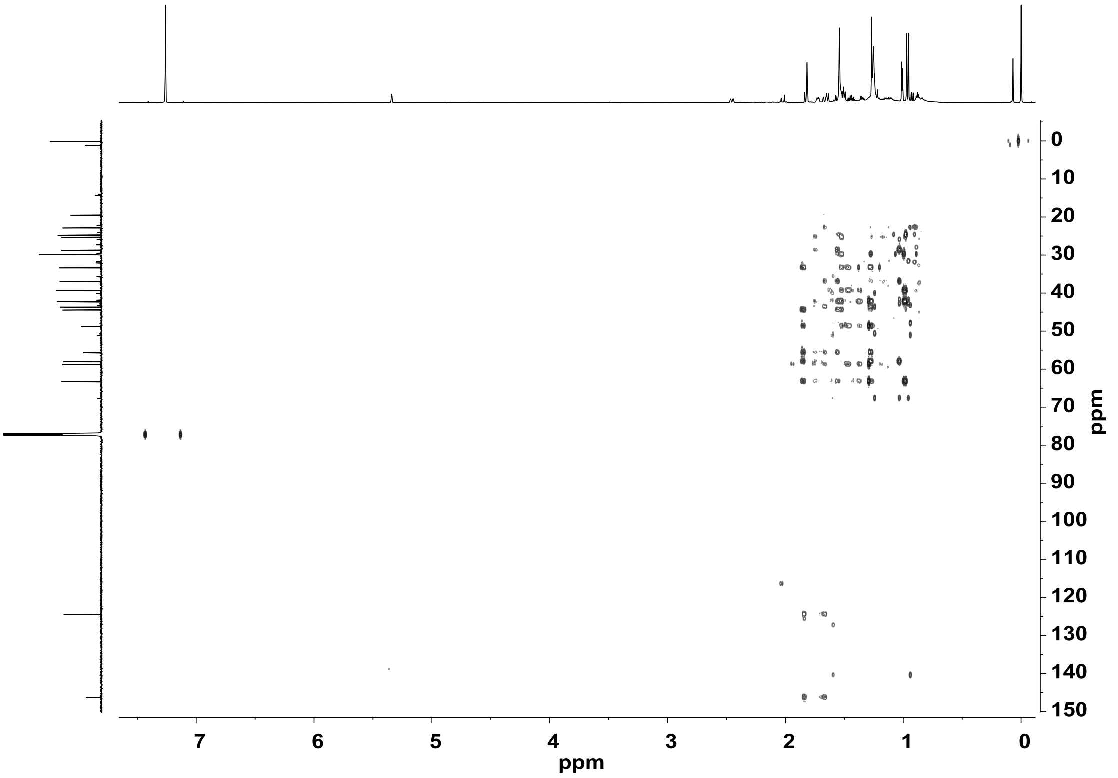

Figure S30. The DEPT 135° spectrum of compound **6** in $CDCl_3$.

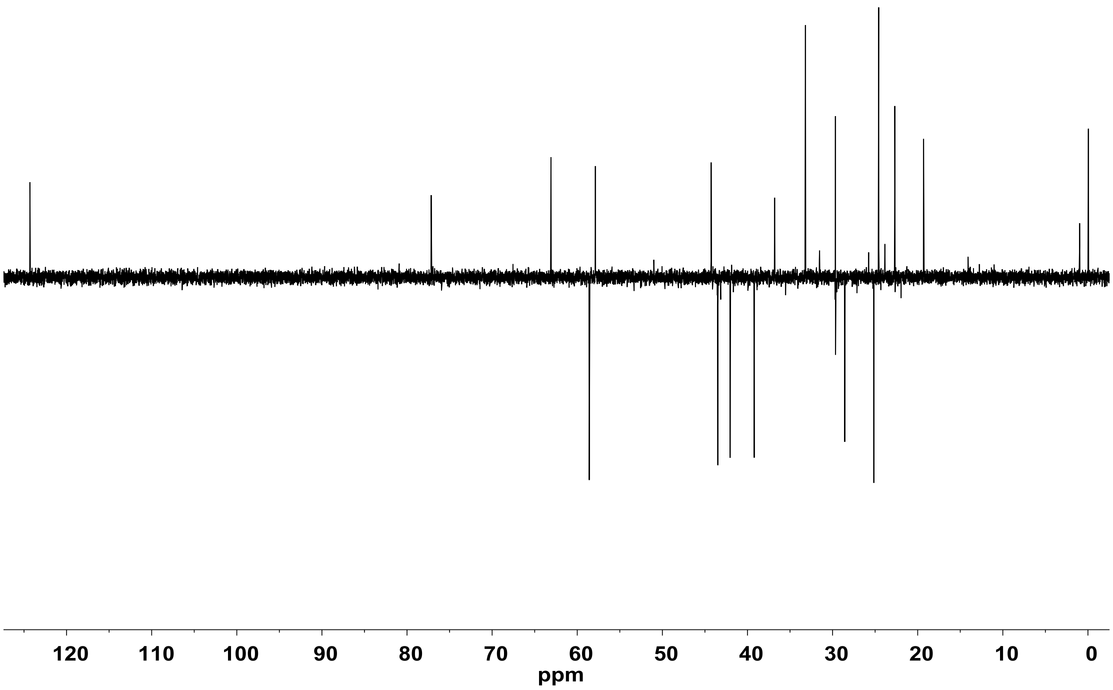

Figure S31. The NOESY spectrum of compound **6** in $CDCl_3$.

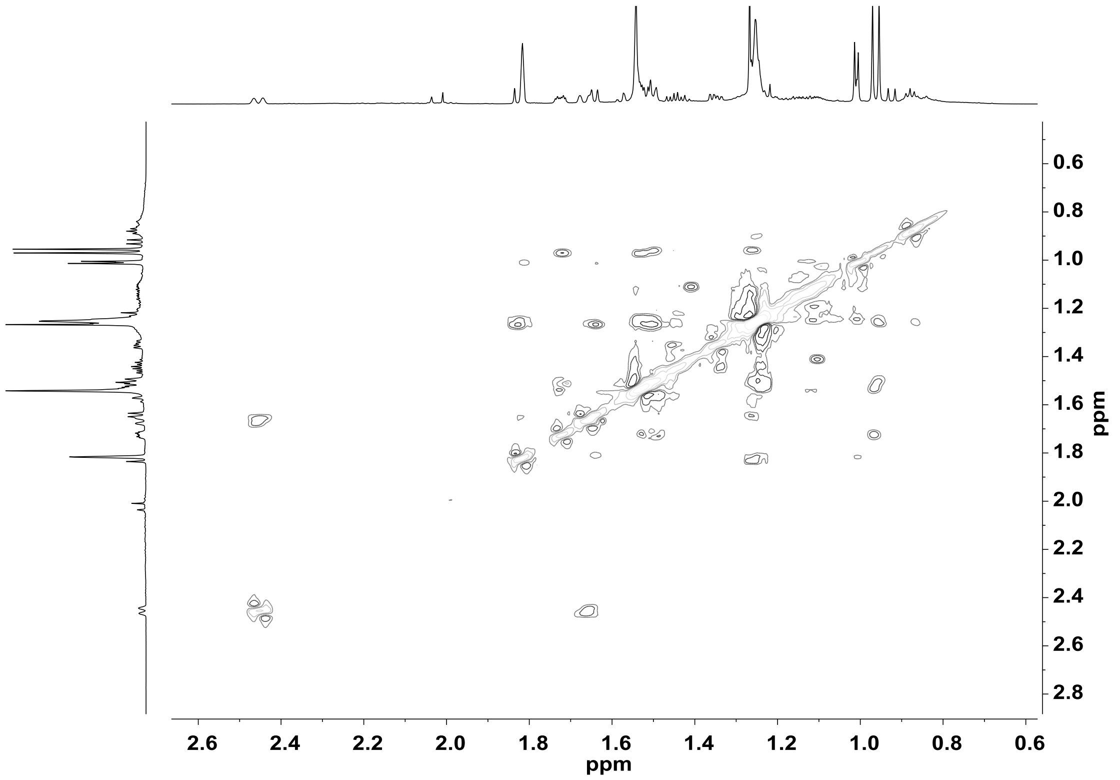

Figure S32. The IR spectrum of compound **6**.

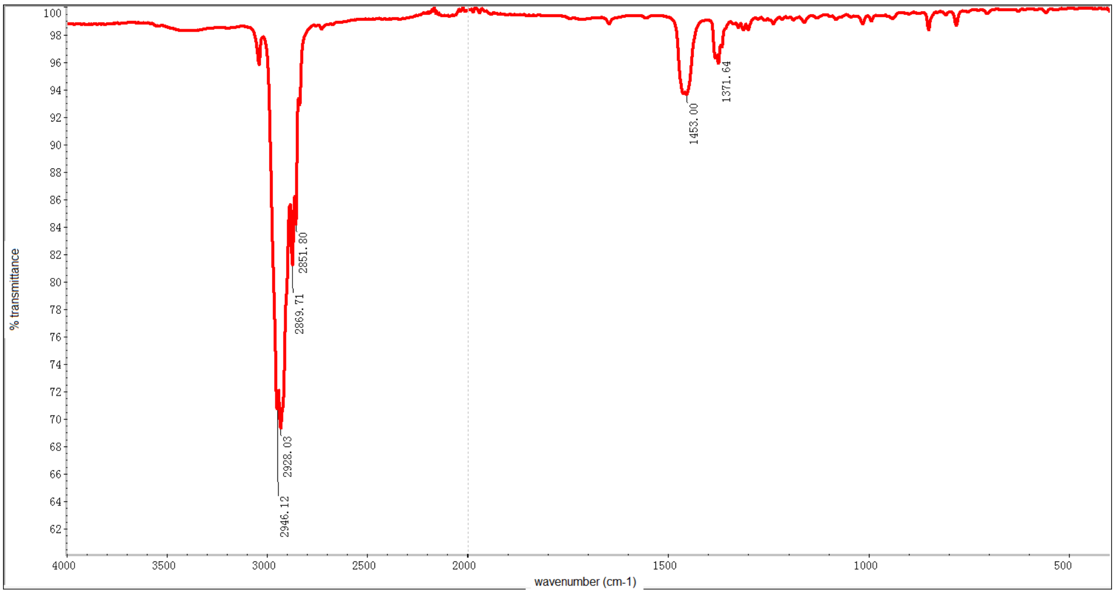

Figure S33. The HRMS (EI) spectrum of compound **6**.

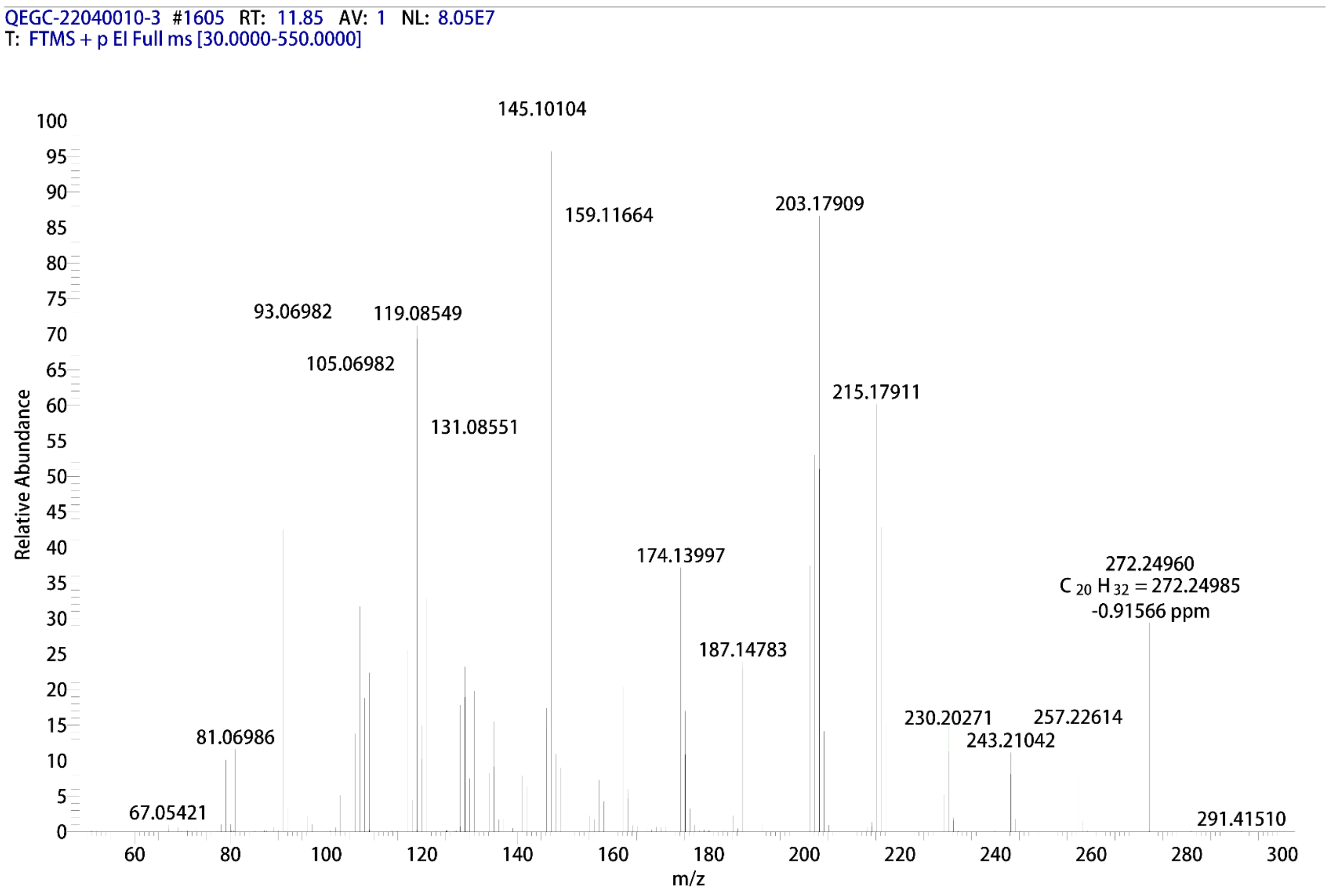

Figure S34. ^{13}C-NMR spectra of A) and J) unlablelled **6**, B) – I) ^{13}C-lableled **6** obtained from the 8 isotopomers of (^{13}C)GGPP labeled at carbons C-1 – C-2 and C-5 – C-10 with CyS^{C59A} and K) – L) ^{13}C-lablelled **6** obtained from the 2 isotopomers of (^{13}C)GGPP labeled at carbons C-3 and C-4 with CyS^{C59A}. Coloured dots correlate the observed ^{13}C signals in the labeling experiments to the individual carbons of **6**. Spectra were measured in C_6D_6 and referenced against solvent signals (^{13}C-NMR: δ = 128.06 ppm).[23]

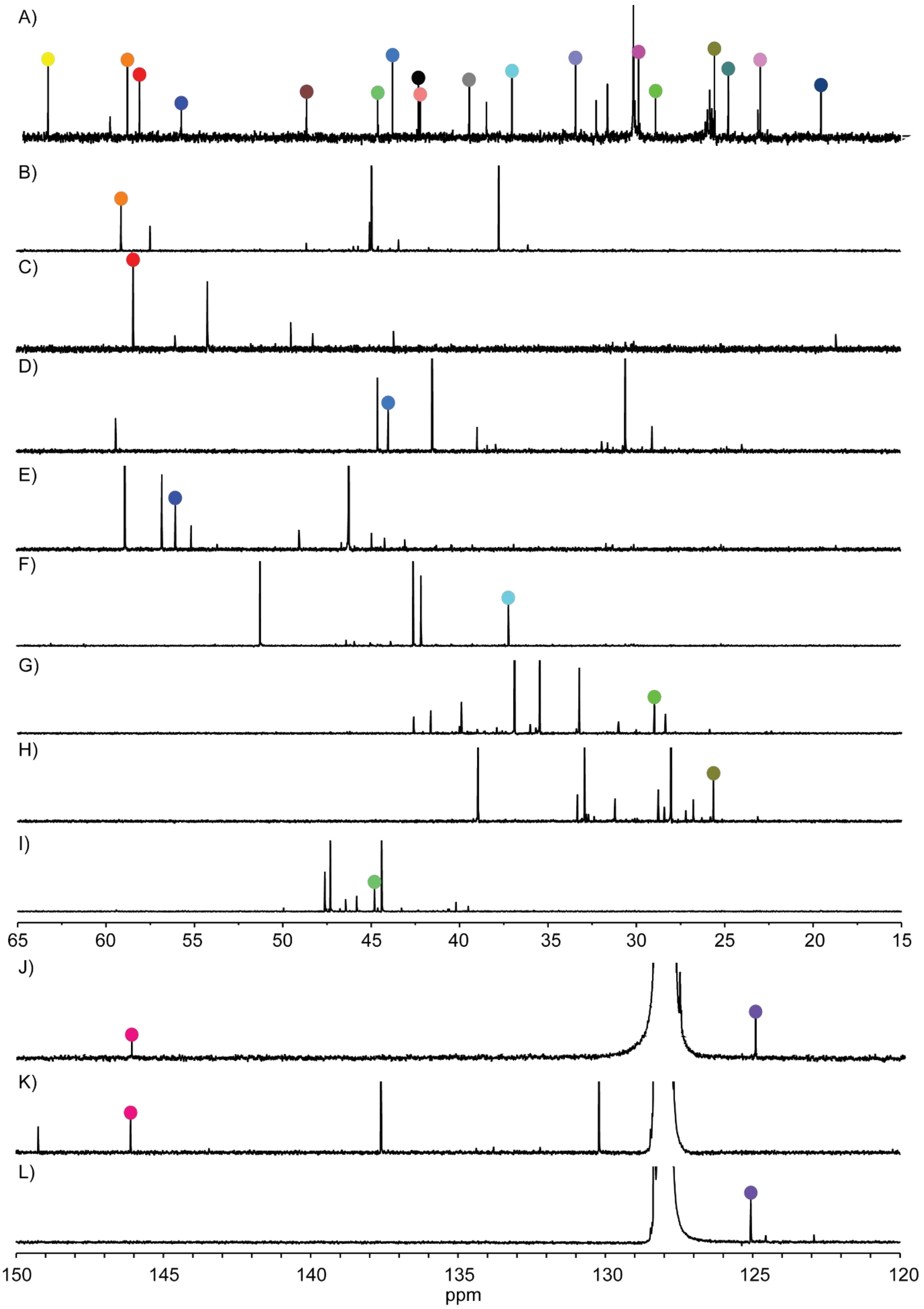

Figure S35. ^{13}C-NMR spectra of A) unlablelled **6** and M) – V) ^{13}C-lablelled **6** obtained from the 10 isotopomers of (^{13}C)GGPP labeled at carbons C-11 – C-20 with CyS^{C59A}. Coloured dots correlate the observed ^{13}C signals in the labeling experiments to the individual carbons of **6**.

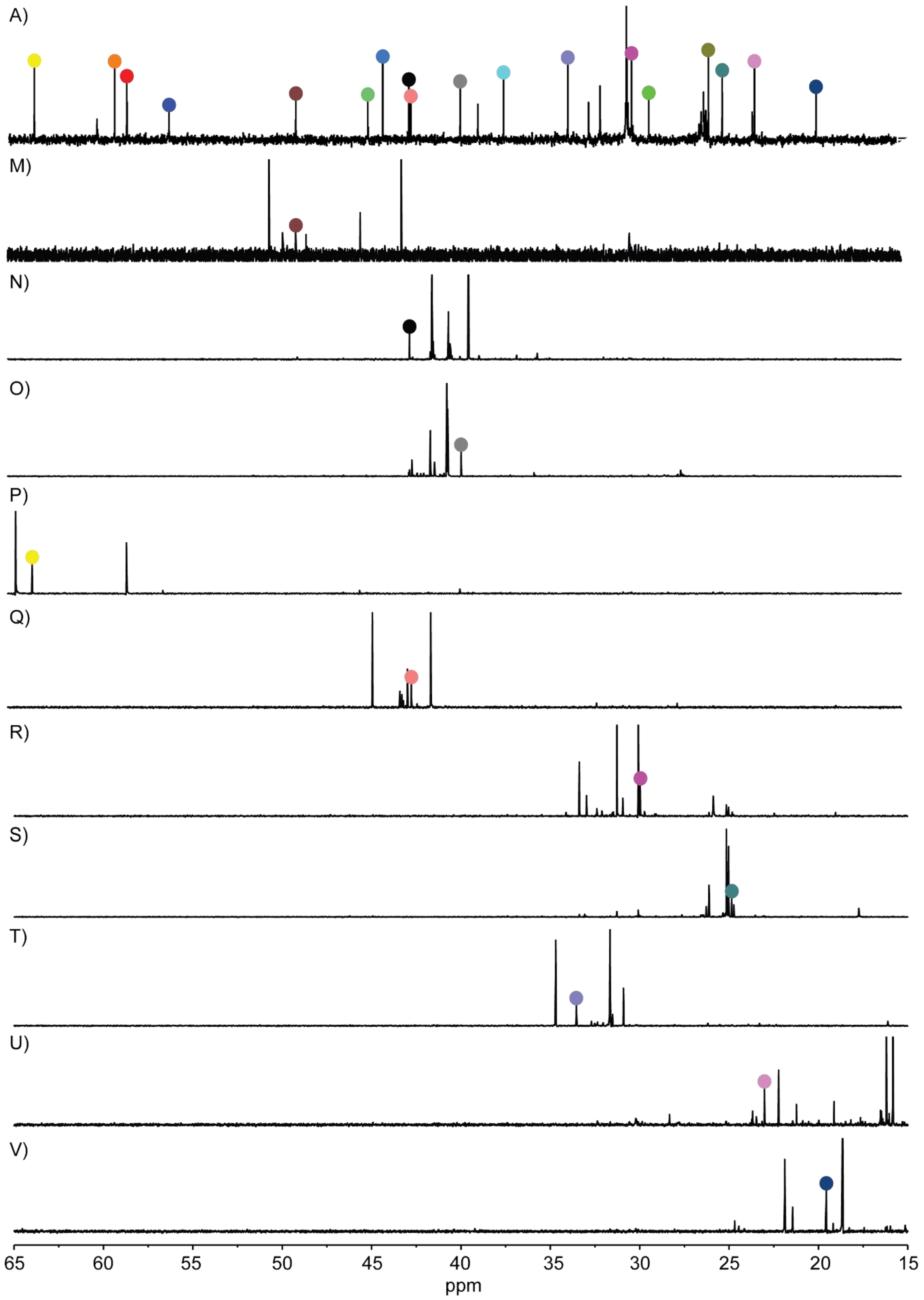

Figure S36. 1,2-Hydride shifts in the biosynthesis of **6**. A) Formation of labeled **6** from (2-^{2}H,3-^{13}C)FPP and IPP with GGPPS and CyS^{C59A}, ^{13}C-NMR spectra (region for C7) of B) (7-^{13}C)-**6** obtained from (7-^{13}C)GGPP with CyS^{C59A}, and C) (7-^{13}C,7-^{2}H)-**6** obtained from (2-^{2}H,3-^{13}C)FPP and IPP with GGPPS and CyS^{C59A}. D) Formation of labeled **6** from (2-^{2}H,3-^{13}C)GGPP with CyS^{C59A}, ^{13}C-NMR spectra (region for C3) of E) (3-^{13}C)-**6** obtained from (3-^{13}C)GGPP with CyS^{C59A}, and F) (3-^{13}C,4-^{2}H)-**6** obtained from (2-^{2}H,3-^{13}C)GGPP CyS^{C59A}. Coloured dots indicate ^{13}C-labeled carbons.

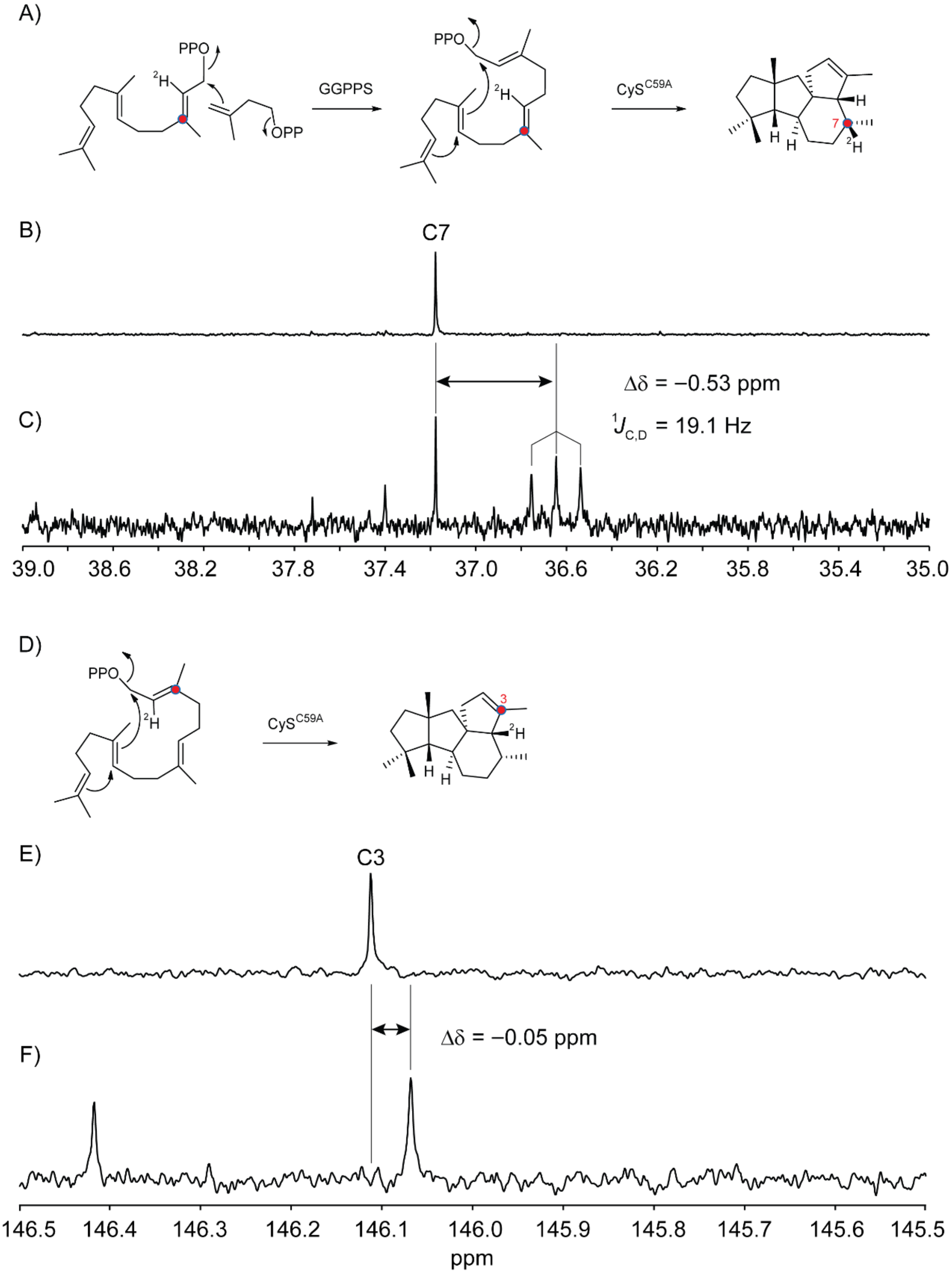

Figure S37. The stereochemical course of the deprotonation to **6**. A) Formation of labeled **6** from DMAPP and (*E*)- or (*Z*)-(4-^{13}C,4-^{2}H)IPP with GGPPS and CyS^{C59A}, ^{13}C-NMR spectra (region for C4) of B) (4-^{13}C)-**6** obtained from (4-^{13}C)GGPP with CyS^{C59A}, C) labeled **6** obtained from DMAPP and (*E*)-(4-^{13}C,4-^{2}H)IPP with GGPPS and CyS^{C59A}, and D) labeled **6** obtained from DMAPP and (*Z*)-(4-^{13}C,4-^{2}H)IPP with GGPPS and CyS^{C59A}. Coloured dots indicate ^{13}C-labeled carbons.

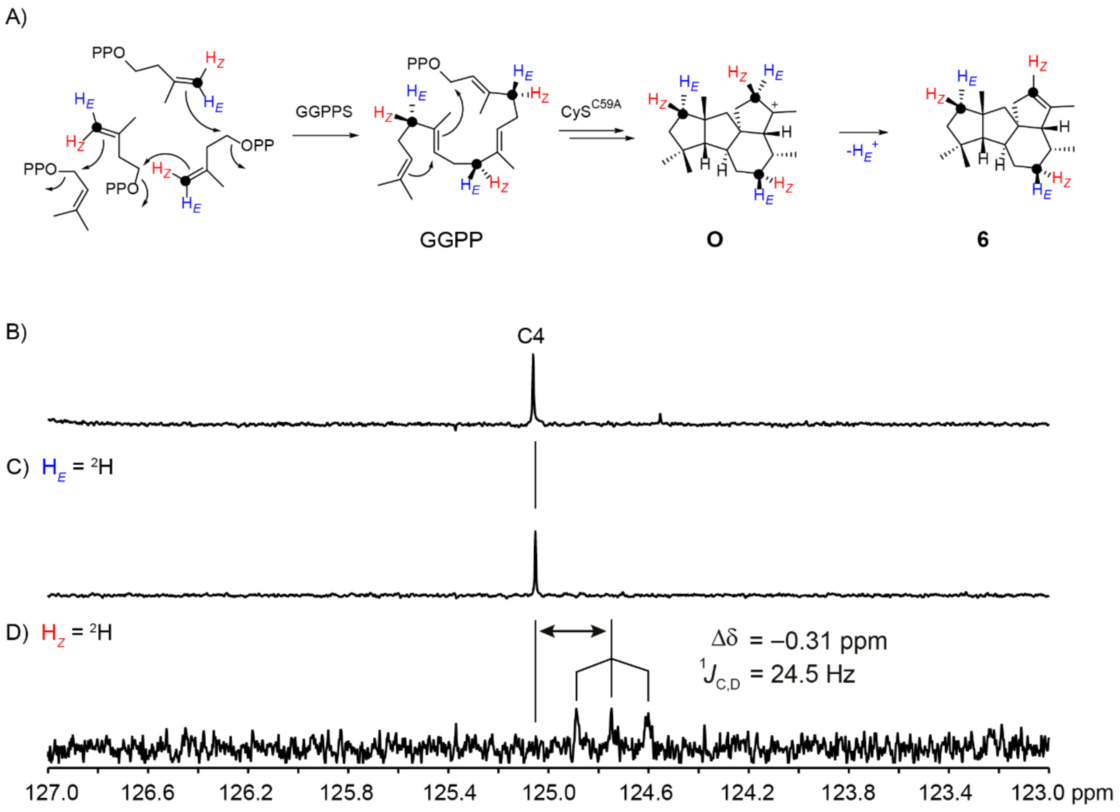

References

[1] Q. S. Wang, K. H. Zhang, Y. Cui, Z. J. Wang, Q. Y. Pan, K. Liu, B. Sun, H. Zhou, M. J. Li, Q. Xu, C. Y. Xu, F. Yu, J. H. He, *Nucl. Sci. Tech.* **2018**, 29, 68.

[2] Z. Otwinowski, W. Minor, *Methods Enzymol.* **1997**, 276, 307-326.

[3] K. Schriever, P. Saenz-Mendez, R. S. Rudraraju, N. M. Hendrikse, E. P. Hudson, A. Biundo, R. Schnell, P. O. Syrén, *J. Am. Chem. Soc.* **2021**, 143, 3794-3807.

[4] T. Weinert, V. Olieric, S. Waltersperger, E. Panepucci, L. Chen, H. Zhang, D. Zhou, J. Rose, A. Ebihara, S. Kuramitsu, D. Li, N. Howe, G. Schnapp, A. Pautsch, K. Bargsten, A. E. Prota, P. Surana, J. Kottur, D. T. Nair, F. Basilico, V. Cecatiello, S. Pasqualato, A. Boland, O. Weichenrieder, B. C. Wang, M. O. Steinmetz, M. Caffrey, M. Wang, *Nat. Methods*. **2015**, 12, 131-133.

[5] A. J. McCoy, R. W. Grosse-Kunstleve, P. D. Adams, M. D. Winn, L. C. Storoni, R. J. Read, *J. Appl. Crystallogr.* **2007**, 40, 658-674.

[6] P. Emsley, B. Lohkamp, W. G. Scott, K. Cowtan, *Acta. Crystallogr. D. Biol. Crystallogr.* **2010**, 66, 486-501.

[7] P. D. Adams, P. V. Afonine, G. Bunkoczi, V. B. Chen, I. W. Davis, N. Echols, J. J. Headd, L. W. Hung, G. J. Kapral, R. W. Grosse-Kunstleve, A. J. McCoy, N. W. Moriarty, R. Oeffner, R. J. Read, D. C. Richardson, J. S. Richardson, T. C. Terwilliger, P. H. Zwart, *Acta. Crystallogr. D. Biol. Crystallogr.* **2010**, 66, 213-221.

[8] A. Rothkirch, G. D. Gatta, M. Meyer, S. Merkel, M. Merlini, H. P. Liermann, *J. Synchrotron. Radiat.* **2013**, 20, 711-720.

[9] O. Trott, A. J. Olson, *J. Comput. Chem.* **2010**, 31, 455-461.

[10] A. O. Chatzivasileiou, V. Ward, S. M. Edgar, *Proc. Natl. Acad. Sci. USA* **2019**, 116, 506-511.

[11] L. A. Johnson, A. Dunbabin, J. C. R. Benton, R. J. Mart, R. K. Allemann, *Angew. Chem. Int. Ed. Engl.* **2020**, 59, 8486-8490.

[12] J. D. Rudolf, L. B. Dong, H. Cao, C. Hatzos-Skintges, J. Osipiuk, M. Endres, C.Y. Chang, M. Ma, G. Babnigg, A. Joachimiak, G. N. Jr. Phillips, B. Shen, *J. Am. Chem. Soc.* **2016**, 138, 10905-10915.

[13] G. Bian, Y. Han, A. Hou, X. Yuan, X. Liu, Z. Deng, T. Liu, *Metab. Eng.* **2017**, 42, 1-8.

[14] L. Lauterbach, J. Rinkel, J. S. Dickschat, *Angew. Chem. Int. Ed. Engl.* **2018**, 57, 8280-8283.

[15] B. Xing, J. Yu, C. Chi, X. Ma, Q. Xu, A. Li, Y. Ge, Z. Wang, T. Liu, H. Jia, J. Guo, L. Huang, D. Yang, M. Ma, *Commun. Chem.* **2021**, 4, 140.

[16] J. Rinkel, S. T. Steiner, J. S. Dickschat, *Angew. Chem. Int. Ed. Engl.* **2019**, 58, 9230-9233.

[17] P. Rabe, J. Rinkel, E. Dolja, T. Schmitz, B. Nubbemeyer, T. H. Luu, J. S. Dickschat, *Angew. Chem. Int. Ed. Engl.* **2017**, *56*, 2776-2779.

[18] J. Rinkel, L. Lauterbach, J. S. Dickschat, *Angew. Chem. Int. Ed. Engl.* **2019**, *58*, 452-455.

[19] P. Rabe, L. Barra, J. Rinkel, R. Riclea, C. A. Citron, T. A. Klapschinski, A. Janusko, J. S. Dickschat, *Angew. Chem. Int. Ed. Engl.* **2015**, *54*, 13448-13451.

[20] G. Bian, J. Rinkel, Z. Wang, L. Lauterbach, A. Hou, Y. Yuan, Z. Deng, T. Liu and J. S. Dickschat, *Angew. Chem. Int. Ed. Engl.* **2018**, *57*, 15887-15890.

[21] A. Hou, J. S. Dickschat, *Angew. Chem. Int. Ed. Engl.* **2020**, *59*, 19961-19965.

[22] T. A. Klapschinski, P. Rabe, J. S. Dickschat, *Angew. Chem. Int. Ed.* **2016**, 55, 10141-10144.

[23] G. R. Fulmer, A. J. M. Miller, N. H. Sherden, H. E. Gottlieb, A. Nudelman, B. M. Stoltz, J. E. Bercaw, K. I. Goldberg, K. I. *Organometallics* **2010**, *29*, 2176-2179.
